# Cannabis use among Arab students: a systematic review

**DOI:** 10.3389/fpsyt.2025.1511563

**Published:** 2025-07-10

**Authors:** Ahmad Sabalbal, Mario Eid, Firas Kobeissy, Evelyne Baroud, Samer El Hayek

**Affiliations:** ^1^ Department of Psychiatry, American University of Beirut Medical Center, Beirut, Lebanon; ^2^ Department of Psychiatry, Lebanese American University, Beirut, Lebanon; ^3^ Center for Neurotrauma, Multiomics & Biomarkers, Department of Neurobiology, Neuroscience Institute, Morehouse School of Medicine, Atlanta, GA, United States; ^4^ Department of Emergency Medicine, McKnight Brain Institute, University of Florida, Gainesville, FL, United States; ^5^ American Center for Psychiatry and Neurology, Dubai, United Arab Emirates

**Keywords:** cannabis, marijuana, Arab, student, school, university

## Abstract

**Introduction:**

The rising global prevalence and potential harms of cannabis use among adolescents and young adults are cause for concern. This systematic review focuses on the Arab world, compiling research on cannabis consumption among school and university students, where use has significantly increased but remains inadequately evaluated.

**Methods:**

The review was registered in PROSPERO (CRD42021285103). Following PRISMA 2020 guidelines, two researchers searched PubMed, Embase, Scopus, and PsycInfo from inception to 9 May 2024, with no filters or language restrictions. Grey literature was identified through structured searches in Google Scholar and ProQuest Dissertations & Theses Global on 30 August 2024, as well as through reference list screening and citation tracking of the included articles. Keywords used included “cannabis”, “student”, and “Arab”.

**Results:**

From 5,820 potentially eligible records, 31 manuscripts were identified and 17 records were retrieved from the grey literature. A total of 48 cross-sectional studies from 13 Arab countries met the inclusion criteria and were included in the synthesis. Of these, 20 studies focused on school settings (sample sizes: 140–10,648), and 29 examined university students (sample sizes: 172–7,445). The most commonly used assessment tools were the Global School-Based Student Health Survey (GSHS) for school students and the WHO-based Alcohol, Smoking, and Substance Involvement Screening Test (ASSIST) for university students. Lifetime cannabis use prevalence ranged from 0.7% in Iraq to 9.4% in Morocco among school students, and from 4.7% in Tunisia to 32% in Lebanon and Egypt among university students. Cannabis use was more prevalent among university students. Key correlates included male gender, older age, family discordance, peer pressure, lower religiosity, and psychiatric symptoms.

**Discussion:**

These findings align with global patterns and emphasize the need for early intervention, psychoeducation, and targeted prevention strategies to mitigate cannabis-related risks among youth in the Arab world.

## 1 Introduction

Cannabis is the most commonly used drug worldwide, especially among young people (1). Some research suggests that it may act as a gateway to other substances (2, 3), while other studies dispute this pathway, emphasizing the role of underlying social, psychological, and environmental factors in substance use progression (4, 5). In 2021, approximately 219 million people used cannabis across nearly all countries and territories, marking a 21% increase from the 180.6 million users in 2011 (6, 7). In 2023, among individuals aged 12 and older, 59.0% (or 167.2 million people) reported using tobacco, nicotine vaping products, alcohol, or illicit drugs. Specifically, 24.9% (or 70.5 million people) used illicit drugs during that period, of which cannabinoids represented 21.8% (or 61.8 million people) (8).

Starting substance use during adolescence is a significant predictor of developing substance use disorders later in life (9). Research using neuroimaging in adolescent substance users has revealed initial evidence of functional and structural alterations in key brain regions, these changes are thought to play a significant role in the future development of substance use disorders (10). Along the same lines, youth who begin to use cannabis before the age of 18 are significantly more likely to develop cannabis use disorder. The risk is highest for those who use cannabis at least weekly, with the highest prevalence found among those who use it daily, and with substance-related issues extending into adulthood leading to negative psychiatric and personal outcomes (11).

In this regard, early cannabis use has been linked to various negative health, social, and behavioral outcomes later in life (12). The former include neurocognitive impairments, such as a decline in verbal intelligence, working memory, and attention (13). These impairments often result in poor academic performance and school dropout. Additionally, young cannabis users tend to associate with peers who engage in substance use or exhibit behavioral problems, exacerbating these issues (14). Other chronic effects include worsening of psychotic disorders in vulnerable individuals, mood disorders, and health conditions affecting the cardiovascular and respiratory systems (15).

This situation is exacerbated by the rising potency of cannabis products and extracts. Cannabis contains over 400 compounds, including more than a hundred cannabinoids that interact with the body’s endocannabinoid system. This system, a critical regulator of the stress response, is made up of neurotransmitters that bind to cannabinoid receptors located throughout the central nervous system (CNS) (16). Among other functions, it is involved in neuroprotection, modulation of nociception, neurogenesis, and pain (17). The most prevalent cannabinoids in cannabis are Δ−9-tetrahydrocannabinol (THC) and cannabidiol (CBD). THC is the primary modulator of the psychoactive effects of cannabis, mainly by binding to the cannabinoid 1 receptors of the CNS (18). Lately, cannabinoid products have displayed significantly elevated levels of THC, worsening their impact on users (19). This has led to a variation in available types of cannabis extracts and concentrates, including Hash, Hash oil, Butane hash oil (BHO), Wax, Dab, Shatter, Amber, and Honeycomb (20), and products specific to the Arab World such as Bango and Hashish which differ by the percentage of cannabinoid constitution (21). Another growing concern is the emergence of synthetic and semi-synthetic cannabinoids. These substances are chemically engineered to act on the same cannabinoid receptors as THC but often produce more potent and unpredictable effects (22). Synthetic cannabinoids, which gained prominence in the early 2000s, are typically structurally unrelated to natural cannabinoids. More recently, semi-synthetic cannabinoids have appeared; these are usually derived from phytocannabinoids and maintain much of their original structure with minor chemical modifications. New variants of both synthetic and semi-synthetic cannabinoids continue to emerge on drug markets worldwide, with considerable variation in their formulations, availability, and prevalence across countries. Like other new psychoactive substances, these compounds cannot be identified using standard toxicology tests (23–25).

The widespread availability and growing potency of cannabis and its related products have global implications, but the situation in the Arab World is particularly concerning. The term “Arab World” refers to a group of 22 countries in the Middle East and North Africa where Arabic is the primary language. The region extends from the Arabian Gulf in the east to the Atlantic Ocean in the west, encompassing countries in both the Middle East and North Africa (26). Although substance use trends vary across the region, the Eastern Mediterranean Region, which significantly overlaps with the Arab World, reports a high burden from substance use disorders, accounting for 4 disability-adjusted life years (DALYs) and 9 deaths per 1,000 population, significantly higher than the global averages of 2 DALYs and 4 deaths per 1,000 population (27). Notably, Egypt, Lebanon, and Morocco stand out as key players in cannabis production and distribution within the Arab World. Since the 1960s, these countries have been the major producers and suppliers of marijuana to worldwide markets (28, 29). While availability is a critical factor in understanding cannabis use trends, recent statistics in the Arab World reveal alarming rates of consumption. In Lebanon, for instance, 56% of young adults aged 18–20 reported lifetime use of cannabis (30). Another study in Egypt found lifetime prevalence reaching 20% among adolescents and young adults (31), while rates reached 13.4% and 11.8% in Morocco and Jordan, respectively (32, 33).

Given the high prevalence of cannabis use and its associated risks, particularly in regions such as the Arab World, understanding the trends, patterns, and factors influencing its consumption is essential. This is especially pressing among young populations who are at heightened risk for early initiation and subsequent substance use (34, 35). As there remains a notable gap in the current literature on cannabis use trends among young people in this region, further investigation among students in the Arab World becomes critical for informing prevention and intervention efforts.

As such, this systematic review aims to synthesize available research on cannabis use among students attending a school or university setting in the Arab World. The review aims to provide valuable insights into the prevalence, trends, characteristics, and correlates of cannabis consumption in this particularly vulnerable population.

## 2 Methods

The protocol of this systematic review was registered in the International Prospective Register of Systematic Reviews PROSPERO (CRD42021285103), in accordance with the Preferred Reporting Items for Systematic Reviews and Meta-Analyses (PRISMA) recommendations for transparency and methodological rigor in systematic reviews.

### 2.1 Eligibility criteria

Studies were eligible to be included in the systematic review if they met the following inclusion criteria:

- Population: Arab adolescents and young adults actively attending a school or university setting in the Arab World (Algeria, Bahrain, Comoros, Djibouti, Egypt, Iraq, Jordan, Kuwait, Lebanon, Libya, Mauritania, Morocco, Oman, Palestine, Qatar, Saudi Arabia, Somalia, Sudan, Syria, Tunisia, the United Arab Emirates, and Yemen).
- Exposure: The use of cannabis and its derivatives, whether current or past.
- Outcome: Prevalence, characteristics, patterns, and correlates of use.
- Study design: Cross-sectional.

Studies assessing the characteristics of cannabis use or looking at overall substance use with stratified data on the consumption of cannabis and its derivatives were included. Studies not directly assessing the above outcomes or where cannabis was described only as a correlate for non-behavioral addictions or mental health conditions were excluded. Studies should have assessed use through a validated instrument or questionnaire; those relying solely on urine drug screening (UDS) were excluded, as UDS does not provide sufficient detail on frequency, context, or patterns of use. Also, studies assessing cannabis use among adolescents or young adults who were neither Arab nor actively enrolled in a school or university setting (i.e., dropouts, refugee populations) were excluded. Furthermore, manuscripts published prior to the year 2000 were excluded, as significant legal, societal, and cultural shifts around that time have likely influenced cannabis use trends, making earlier data less comparable to more recent findings (36). Other types of quantitative studies, reviews, correspondences, case reports or series, opinions, letters to the editor, and book chapters were excluded. Lastly, studies reported as abstracts for which the authors could not identify a full text after contacting the corresponding author were excluded.

### 2.2 Search strategy

PubMed, Embase, Scopus, and PsycInfo databases were searched from inception to 9 May 2024. No filters or language restrictions were applied. The MeSH terms and search strategy are outlined in the **Supplementary Material**. To identify grey literature, on 30 August 2024, the authors conducted structured searches using the keywords *(cannabis OR marijuana) AND student AND Arab* in Google Scholar and ProQuest Dissertations & Theses Global (https://www.proquest.com/), screening the first 50 pages of results. The authors also hand-searched the reference lists of all the included studies and used the “cited by” function in Google Scholar to identify relevant citing articles.

### 2.3 Selection process

All studies obtained through the literature search were imported using EndNote software version 21. After the removal of duplicate articles, two reviewers (AS and SEH) did an independent title and abstract screening of the retrieved studies using a standardized screening guide. The same reviewers then assessed the eligibility of the resulting group of articles using a full-text screening guide. Any disagreement was resolved by discussion. Details of both screening guides are provided in the **Supplementary Material**.

### 2.4 Data extraction

Two reviewers (AS and SEH) independently abstracted the following data from each eligible study: objectives, sample characteristics (recruitment site, sample size, age, and gender distribution), instruments used to assess cannabis use, prevalence of cannabis use, characteristics of users, and cannabis-related outcomes or correlates. Discrepancies were resolved through discussion.

### 2.5 Bias assessment

To evaluate the methodological quality and risk of bias of the included studies, The National Institutes of Health (NIH) Quality Assessment Tool for Observational Cohort and Cross-Sectional Studies was used (37) (**Supplementary Material**). The tool consists of a 14-question checklist designed to assess the internal validity of cross-sectional and cohort studies. It addresses potential risks related to selection, information, or measurement bias, as well as confounding factors. Each criterion was evaluated with responses of “yes”, “no”, “not applicable”, or “not reported.” According to the quality rating guidance provided with the tool, each included study was rated as good, fair, or poor quality. Two researchers (AS and SEH) independently assessed the final studies using the tool, and any disagreements were resolved through discussion until consensus was achieved.

### 2.6 Data analysis

Although the authors originally planned for a quantitative synthesis of the results, it was not possible to conduct it due to the heterogeneity of the included studies. Instead, a qualitative synthesis of the data was performed.

## 3 Results

**Figure 1:**
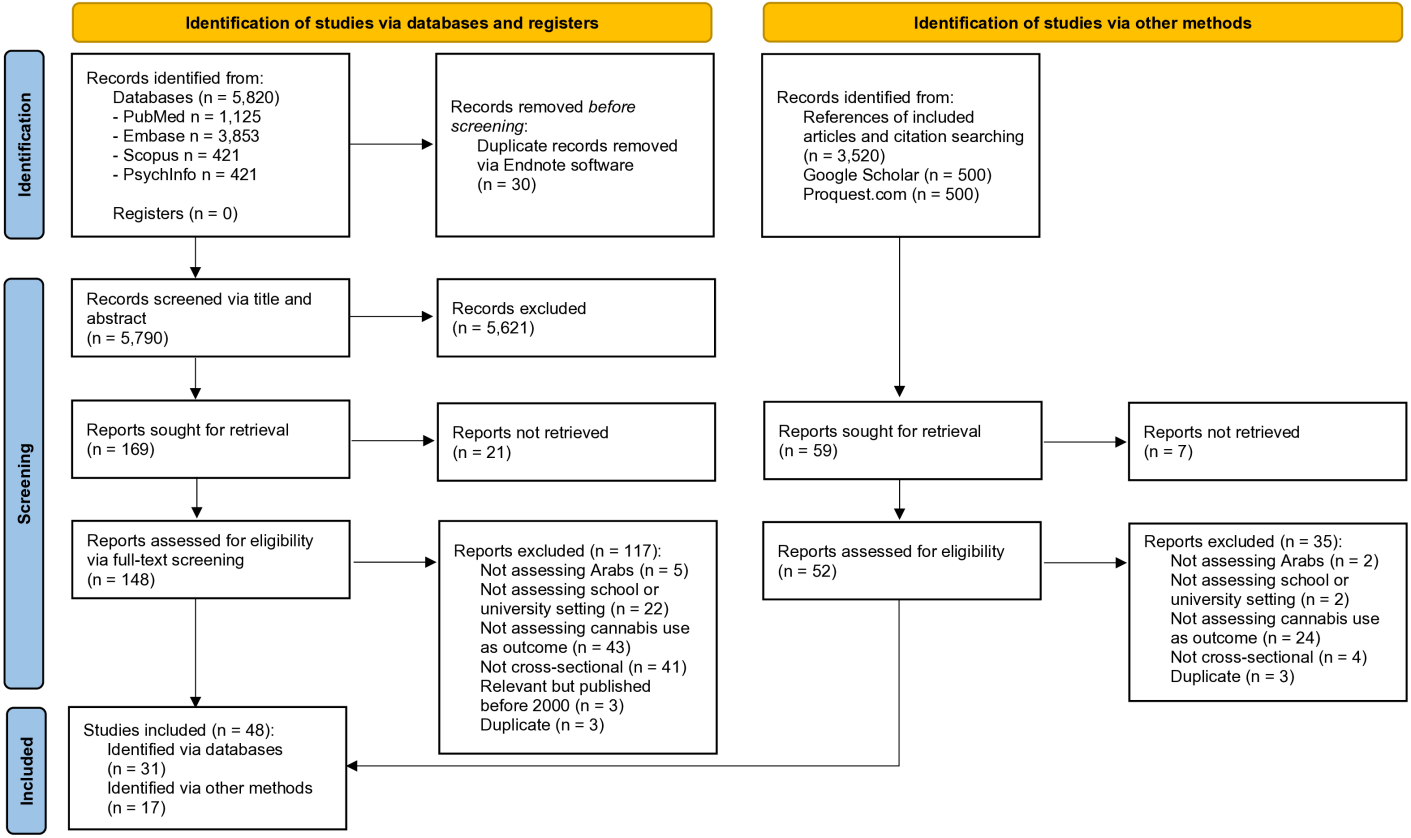
PRISMA 2020 flow diagram for the systematic review.

A total of 5,820 potentially eligible records were obtained after the electronic search of the databases (i.e., PubMed, Embase, Scopus, and PsycInfo). After the removal of duplicates, 5,790 records were processed via an initial title and abstract screening, of which 5,621 records were excluded. Of the remaining 169 articles, 138 were excluded after full-text screening for the reasons outlined in the PRISMA Flow Diagram (**Figure 1**). In addition to the 31 manuscripts identified via databases, 17 additional records were obtained via other methods (i.e., grey literature sources, which included Google Scholar and ProQuest Dissertations & Theses Global, references of included articles, and citation tracking). In total, 48 studies were included in the narrative synthesis. Participants were university students (n=29) or school students (n=20). One study recruited both university and school students (38). Data emanated from 13 Arab countries, mainly Egypt (n=13), Lebanon (n=8), Morocco (n=8), Palestine (n=7), Kuwait (n=5), Tunisia (n=5), Jordan (n=3), Algeria (n=2), Iraq (n=2), Mauritania (n=1), Oman (1 study), Saudi Arabia (n=1), and Sudan (1 study). Most studies were rated as fair or good according to the NIH Quality Assessment Tool. The results of the bias assessment are depicted in **Tables 1**, **2**.

**Table 1:** Studies exploring cannabis use among students attending a school setting (n=20).

Study	Objectives, study site, and country	Sample characteristics	Instrument to assess cannabis use	Prevalence of use	Associations and correlates of use	Risk of bias
Al-Musa and Al-Montashri (39)	Determine the prevalence of lifetime substance use and its correlates. One randomly chosen secondary school in Abha city, Saudi Arabia.	**Sample size:** n=400 **Age:** Range: <17: 19.8% 17–18: 68.8% >18: 21.5% **Gender:** 100% males	Questionnaire adapted from the State and Local Youth Risk Behavior Survey (YRBS) and the Global School-Based Student Health Survey (GSHS)	*Illicit substance use: 8.8% Among substance users, 51.4 % were cannabis users.	Among illicit substances, cannabis was the most commonly used substance.	Fair
Alzyoud (40)	Determine the patterns and risk factors of cannabis use. Three school districts in Zarka, Jordan.	**Sample size:** n=384 **Age:** Mean 15.53 ± 1.324; Range 13-18 **Gender:** 50.3% males	Cannabis Abuse Screening Test (CAST) Adolescent Knowledge, Attitude, and Beliefs about Substance Use Questionnaire (AKABSU)	No cannabis prevalence data were provided. **11.7% (n=45) answered the CAST:** -Most were males (n=37) -Most started using at ages 16 (n=19) or 17 (n=17) -The youngest age to start using was 13 (n=2) -22 reported smoking alone -28 reported a problem due to cannabis use -27 reported memory problems due to cannabis use -19 attempted to stop cannabis and failed -14 scored a problematic cannabis use	**Among those who answered the CAST:** *Cannabis use was more common: -In males compared to females (χ^2^ = 20.690, OR=5.117, p=0.0001). -In those having family problems (χ²=25.087, OR=19.396, p=0.0001). *Cannabis use was less common: -In those living with both parents (χ²=23.375, OR=0.159, p=0.0001). *There was a positive relationship between cannabis use and: -Scoring ≥4 points on the mental health subscale, indicative of a high-risk mental status problem (χ²=22.522, p=0.0001). -Having poor social skills (r=0.304, p=0.0001). -Having positive attitudes towards cannabis use (r=0.637, p=0.0001). -Having the belief that cannabis use helps in forming friendships (r=0.413, p=0.0001). **Among all participants:** -77.1% had heard about cannabis use. -31.8% knew what cannabis use is and identified it as smoking cannabis. -30.8% reported knowing the consequences of cannabis use, such as developing a habit or addiction. -61.2% were unaware of the problems associated with cannabis use. -75.5% indicated that they would not participate in cannabis use with a group of friends who use cannabis. -69.8% viewed cannabis use as a problem. -80.0% indicated that cannabis use goes against society norms. -81.3% believed that adolescent cannabis use is a problem.	Fair
**Azaiza et al. (** 41)	Examine rates of psychoactive substance use and links with sociodemographic, interpersonal, and personality characteristics. Schools (grades 7 to 12) located in Arab and mixed villages, Palestine.	**Sample size:** n=2,603 **Age:** Range 12-18 **Gender:** 45.53% males	Self-developed questions	**Past year, month, and week use, respectively:** Cannabis: 5.2%, 5.2%, 4.8% Hashish: 3.2%, 3.0%, 2.7% Marijuana: 4.5%, 4.3%, 4.1%	**Past year cannabis use was more common:** -Among males compared to females (*χ* ^2^ = 80.9, p<0.001). -Among low religiosity compared to medium/high religiosity groups (*χ* ^2^ = 29.91, p<0.001). -Among those who perceived their family as less coherent (i.e., more separated) (χ^2^ = 26.82, p<0.001). -Among those with higher behavioral intention (χ^2^ = 70.91, p<0.010) and risk perception (χ^2^ = 21.78, p<0.010). **Past year cannabis use was less common:** -Among those living with both parents compared to those living in any other arrangement (χ^2^ = 6.68, p<0.010). -Among those with higher sense of coherence (χ^2^ = 15.83, p<0.001) and hardiness (χ^2^ = 15.85, p<0.001).	Fair
**Azaiza et al. (** 42)	Examine trends in psychoactive substance use. Sixty schools (grades 7 to 12) in the Galilee, the Triangle, and the Negev, Palestine.	**Sample size:** n=2,944 **Age:** Range 12-18 **Gender:** 39.75% males	Self-developed questions	**Past year, month, and week use, respectively:** Cannabis: 6%, 5.8%, 5.8% Hashish: 5.4%, 5.2%, 4.9% Marijuana: 4.9%, 4.8%, 4.7%	**Significant predictors of past year cannabis use:** -Gender (β=1.22, p<0.001) -Positive attitudes (β=0.81, p=0.001) -Family cohesion (β=-0.41, p=0.010) -Risk perception (β=-0.36, p=0.001) **Past year cannabis use was more common:** -Among males compared to females (*χ* ^2^ = 101.13, p<0.001). -Among low religiosity compared to medium/high religiosity groups (*χ* ^2^ = 38.15, p<0.001).	Fair
**Azaiza et al. (** 43)	Examine rates of psychoactive substance use and correlates of use. Fifteen schools (grades 7 to 12) in Palestine.	**Sample size:** n=519 **Age:** Range 12-18 **Gender:** Not specified	Self-developed questions	**Past year, past month, and past week use, respectively:** Cannabis: 3.7%, 3.3%, 3.3% Hashish: 3.1%, 2.7%, 2.5% Marijuana: 3.6%, 2.9%, 2.6%	**Significant predictors of past year cannabis use:** -Gender: Males 8.2 %, Females 0.3% (*χ* ^2^ = 21.74, p<0.001) -Religiosity: Low 7%, Medium 1.6%, High 2.8% (*χ* ^2^ = 9.59, p<0.01)	Fair
Damiri et al. (44)	Provide insight into the extent of substance use and patterns of use. Schools in the West Bank, Palestine.	**Sample size:** n=828 **Age:** Range 15-16 **Gender:** 48.4% males	Questionnaire adapted from the Monitor the Future Study and the European School Survey Project on Alcohol and Other Drugs (ESPAD)	**Lifetime cannabis use:** Marijuana: 2.5% Hashish: 2.2% **Lifetime cannabinoids use:** 12.8% Mr. Nice guy: 3.0% Bango: 2.7% Mabsoton: 2.6% Spice: 2.5% Hydro: 2.2% Mastalon: 1.8%	Both natural and synthetic cannabinoids were the most frequently (19.5%) ever used illicit drugs. Most students (74.7–97.3%) heard about hashish and marijuana while less (41.1–55.5%) heard about synthetic cannabinoids.	Good
Diamond et al. (45)	Estimate the prevalence of drug and alcohol use. Students from two high schools (grades 9 to 12) in the Bedouin of the Negev, Palestine.	**Sample size:** n=440 (295 high school students and 145 school attriters) **Age:** Range 14-18 **Gender:** 52% males	Questionnaire adapted from the National Epidemiologic Survey on Alcohol and Related Conditions (NESARC)	Among the students group: **Past year cannabis use:** 18% **Past year hashish use:** 16.4% **Past year marijuana use:** 16.4%	**Significant predictors of past year cannabis use** (combined data for students and school attriters): -Gender: Males 22%, Females 8.1% (*χ* ^2^ = 14.34, p<0.001) -Age: -14: 19%, 15-16: 21.9%, 17+: 8.5% (*χ* ^2^ = 15.84, p<0.001) -Religiosity: Secular: 45.3%, Devout 13%, Practicing: 10.3% (*χ* ^2^ = 37.86, p<0.001)	Good
El Omari et al. (46)	Evaluate the use of psychoactive substances. Eighty randomly chosen schools in Morocco.	**Sample size:** n=2,603 **Age:** Mean 17.1 ± 1.5; Range 15-17 **Gender:** 46% males	Questionnaire adapted from the ESPAD	**Lifetime cannabis/hashish use:** Total: 9.4% Males 9.5%; Females 2.1% **Past year cannabis/hashish use:** Total: 5.7% Males 6.4%; Females 0.7% **Past month cannabis/hashish use:** Total: 4.7% Males 5.8%; Females 0.6%	-Most students initiated cannabis/hashish use at the age of 15 (no percentage reported). -Among past month users, most (38.7%) used cannabis/hashish for 2–5 days while 12.3% used it for more than 20 days. *Significant predictors of cannabis use: -Being absent from school (p<0.001) -Having low grades on the last trimester -Sleeping outside home (p<0.001) -Easy access for substances (p<0.001) -Having a family member or friend who consumed alcohol (p<0.001) -Perceiving cannabis as harmless (p<0.001) 91% of participants reported good knowledge about hashish and related products.	Good
**Karam et al. (38)	Describe the extent and patterns of alcohol and illicit substances use. Ten private schools and two of the largest private universities in Lebanon.	**School sample:** **Sample size:** n=1,307 **Age:** Mean 17.1 ± 0.9 **Gender:** 51.8% males	Diagnostic Interview Schedule version IV, based on the Diagnostic and Statistical Manual of Mental Disorders IV (DSM-IV) criteria	**Lifetime Hashish/Marijuana use in school sample:** Total 6.8% Males 9.1%; Females 4.3%	**Among the school sample:** -Hashish/marijuana was the most tried illicit substance. -Lifetime use was significantly higher in males compared to females (p<0.005). -Age of onset of ever using cannabis: 16.1 ± 1.5 -57.3% thought that the use of hashish/marijuana was a crime. -8.6% thought that the use of hashish/marijuana would lead to the consumption of “harder” drugs. -26.6% thought that hashish/marijuana were as bad as cocaine/heroin.	Good
Loffredo et al. (47)	Determine the prevalence and correlates of substance use. Twenty-five public schools in Egypt.	**Sample size:** n=1,415 **Age:** Range 12-18 **Gender:** 72% males	Self-developed questions (with questions selected from previously validated surveys that addressed substance use)	**Lifetime hashish use:** Males: 6.2%; Females: 4.3% **Lifetime Bango use:** Males: 5%; Females: 0% **Past month mean number of days of Hashish use:** Males: 8.5 ± 9.4; Females: 6.5 ± 4 **Past month mean number of days of Bango use:** Males: 6.1 ± 8.2	**Age at initiation of use:** -Hashish: Males 15.1 ± 1.6; Females 15.8 ± 1.1 -Bango: Males 15.5 ± 2.0 **Lifetime hashish use was significantly higher in:** -Males compared to females: OR=2.08, CI 1.04-4.18. -Those working: OR=1.96, CI 1.14-3.38 -Those not living with parents: OR=2.93, CI 1.35-6.35. **Lifetime hashish use was significantly lower in:** -Younger age groups (12–14 years: OR=0.14, CI 0.04-0.41 and 14–16 years: OR=0.31, CI 0.17-0.59) compared to the group >16 years. -Those residing in Sohag and Qena (southern areas of Egypt) compared to those residing in Cairo: OR=0.37, CI 0.17-0.79. **When looking at male data only:** **Lifetime hashish use was significantly higher in:** -Those working: OR=1.99, CI 1.06-3.72. **Lifetime hashish use was significantly lower in:** -Younger age groups (12–14 years: OR=0.16, CI 0.05-0.47) compared to the group >16 years. -Those residing in Sohag and Qena (southern areas of Egypt) compared to those residing in Cairo: OR=0.11, CI 0.03-0.41.	Good
Mahmood et al. (48)	Determine the prevalence of substance use and associated factors. Thirty randomly selected schools in Erbil City, Iraq.	**Sample size:** n=2,743 **Age:** Mean 16.7 ± 1.249; Range 14-19 **Gender:** 56.3% males	Questionnaire adapted from the School Survey on Drug Use from the United Nations Office on Drugs and Crime (UNODC)	**Lifetime marijuana/hashish use:** 0.7% **Past year marijuana/hashish use:** 0.3% **Past month marijuana/hashish use:** 0.3%	None.	Good
Omu et al. (49)	Establish information about teenagers’ substance use, awareness of peer use, and knowledge regarding health risks of addiction. School (grades 9 to 12) in Kuwait.	**Sample size:** n=190 **Age:** Range 15-18 **Gender:** 59.5% males	Adopted from the 144-item 1998 New Jersey Triennial Public High School Survey of Drug and Alcohol Use questionnaire	**Lifetime Hashish/Marijuana use:** 5.3% **Current Hashish/Marijuana use:** 3.7%	-Hashish was the most common ever and currently used illicit substance. -Hashish users reported that they get their supply from friends and use them in their rooms or in parks, either alone or with friends. -Hashish users reported to have tried to stop at least once without success. **Among all participants:** -70% and 54.7% said that they were aware of the physical and mental health risks of hashish, respectively. - Males were more knowledgeable about the physical (U=3340.500, p=0.001) and mental (U=3085.000, p=0.000) changes associated with Hashish use than females. -Males believed that there has been an increase in Hashish use (U=3636.000, p=0.043).	Good
Peltzer and Pengpid (50)	Assess the prevalence of cannabis use and associated factors. Schools (grades 6 to 10) in Algeria, Morocco, and Palestine, among other countries.	**Sample size:** n=15,226 (29.8%, 19.2%, and 17.6% from Algeria, Morocco, and Palestine, respectively) **Age:** Range 13-16 **Gender:** 50.8% males	GSHS	**Lifetime cannabis use:** Algeria: 2.3% (1.5-3.0) Morocco: 3.7% (2.5-4.9) Palestine: 3.9% (2.3-5.4) **Past month cannabis use:** Algeria: 2.2% (1.3-3.0) Morocco: 2.6% (1.6-3.5) Palestine: 4.1% (2.3-5.9)	None.	Good
Peltzer and Pengpid (51)	Estimate the prevalence of lifetime cannabis and amphetamine use and associated factors. Schools from Iraq and Kuwait, among other countries.	**Sample size:** Iraq: n=2,038; Kuwait: n=2,672 **Age** (for the total sample): Mean 15.4 **±** 1.5; Range 11-18 **Gender:** Not mentioned	GSHS	**Lifetime cannabis use:** Iraq: Total 2.4% Males 3%; Females 1.6% Kuwait: Total 3.2% Males 5.7%; Females 0.4% **Past month cannabis use:** Iraq 3.1%; Kuwait 2.2%	**Factors associated with lifetime cannabis use in Kuwait:** -Male gender: OR=11.17, CI 2.97-42.02, p<0.001 -Anxiety: OR=2.48, CI 1.19-5.20, p<0.050 -Suicidal ideation: OR=3.91, CI 1.63-9.35, p<0.010 -Current smoking: OR=7.30, CI 2.34-22.73, p<0.010 -Parental/guardian tobacco use: OR=2.13, CI 1.30-3.49, p<0.010 **Factors associated with lifetime cannabis use in Iraq:** -Current smoking: OR=9.20, CI 3.62-23.41, p<0.001 -School truancy: OR=0.35, CI 0.17-0.71, p<0.010 -Parental/guardian tobacco use: OR=2.59, CI 1.04-6.47, p<0.050 -Food insecurity: OR=9.77, CI 3.03-31.56, p<0.001	Good
Peltzer and Pengpid (52)	Estimate the prevalence of cannabis and amphetamine use and associated factors. Schools from Algeria, Mauritania, and Morocco (classes of year 1 to 4 in secondary schools), among other countries.	**Sample size:** Algeria: n=4,532; Mauritania: n=2,063; Morocco: n=2,924 **Age** (for the total sample): Mean 14.3 **±** 1.6 **Gender:** Not mentioned	GSHS	***Past month cannabis use: Algeria: Total 2.2% Males 4%; Females 0.4% Mauritania: Total 6.9% Males 7%; Females 6.3% Morocco: Total 2.6% Males 3.9%, Females 0.7%	In both Algeria and Morocco, males were significantly more likely to use cannabis than females (no p-value provided).	Good
Rabie et al. (53)	Detect the prevalence of substance use and dependence. Randomly selected secondary classes from several schools in three Governorates in Egypt.	**Sample size:** n= 10,648 **Age:** Range 13-18 **Gender:** 31.7% males	Questions adapted from the Addiction Severity Index Scale	**Lifetime, past year, and past month use, respectively:** Cannabis: 2.7%, 2.6%, 1.6% Vodo: 1.6%, 1.2%, 0.8% **Cannabis dependence (as per DSM-IV criteria):** Cannabis: 0.5% Vodo: 0.27%	After exclusion of tobacco, cannabis was the third most commonly used substance during the past year (following alcohol and organic solvents). The prevalence of cannabis dependence was the highest compared to all other illicit substances. Lifetime, past year, and past month cannabis use were higher in males compared to females (p-value not provided).	Good
Shaheen et al. (54)	Assess the prevalence of substance use and associated risky behaviors. Two schools in Dragil village, Egypt.	**Sample size:** n=140 **Age**: Mean 16.46 ± 0.51 (among n=26 substance users) **Gender:** 65.7% males	Questionnaire adapted from the World Health Organization on health risky behaviors	*Types of used substances: Hashish/cannabis: 73.1% Bango: 42.3% Strox: 3.8% Voodoo: 3.8%	The most used illicit substance was hashish/cannabis followed by Bango.	Poor
Soliman et al. (55)	Explore the association between substance abuse and part-time work among vocational students. Five vocational schools in Menoufia, Egypt.	**Sample size:** n=316 **Age:** Mean 17.27 ± 1.11; Range 15-19 **Gender:** 100% males	Self-developed questions	**Past month cannabis use:** 24% **Past month marijuana use:** 16.4%	After tobacco, cannabis and marijuana were the second and third most commonly used substances, respectively, in the past month. In contrast to day-only and day/night workers, those who worked only at night had significantly lower past month use of cannabis (p<0.001) and marijuana (p<0.001).	Fair
Zammit et al. (56)	Determine the prevalence and factors associated with illicit substance use. Sixteen public middle schools (grades 7 and 9) in the region of Sousse, Tunisia.	**Sample size:** n=4,272 **Age:** Mean 13.3 ± 1.2 **Gender:** 49.5% males	Self-developed questions	**Lifetime cannabis use:** 1.9%	Among illicit substances, cannabis was the most commonly ever used substance.	Good
Zarrouq et al. (57)	Estimate the prevalence and determinants of cannabis and other substance use. Public middle and high schools in the North central region of Morocco.	**Sample size:** n=3,020 **Age:** Mean 16 ± 2; Range 11-23 **Gender:** 53% males	Self-developed questions	**Lifetime cannabis use:** Total 8.1% Males 13.5%; Females 1.9% **Past year cannabis use:** Total 6.7% Males 11.6%; Females 1.1%, **Past month cannabis use:** Total 5.6% Males 9.8%; Females 0.9%,	Among illicit substances, cannabis was the most commonly ever used substance.	Good

*The type of measured prevalence (i.e., lifetime, past year, etc.) is not specified; **this study is also displayed in **Table 2** **;** ***Same data set as Peltzer and Pengpid (50) but with additional information, including for Mauritania.

**Table 2:** Studies exploring cannabis use among students attending a university setting (n=29).

Study	Objectives, study site, and country	Sample characteristics	Instrument to assess cannabis use	Prevalence of use	Associations and correlates of use	Risk of bias
Abdelmounaim et al. (58)	Assess the prevalence of substance use among students and compare patterns to a sample of non-students. Cadi Ayyad University, Morocco.	Analysis included n=1,054 participants, of whom n=444 were students. **Students group:** **Sample size:** n=444 **Age**: Mean 22.15 **±** 2.79; Range 17-30 **Gender:** 87.12% males	Self-developed questions	No cannabis prevalence data were provided. **Among the students group:** -15.5% reported cannabis to be their first used substance (ranked second after tobacco). -29.1% reported cannabis as their principle substance (ranked second after tobacco).	**Among the students group:** In polysubstance users, 8.1% used cannabis and tobacco (ranked second after alcohol and tobacco).	Poor
Alsammak et al. (32)	Assess substance use and explore potential risk factors. University of Jordan, Hashemite University, and Jordan University of Science and Technology, Jordan.	**Sample size:** n=1,184 **Age:** Mean 20.89 **±** 2.347 **Gender:** 38.4% males	Questionnaire adapted from the World Health Organization guidelines	Of 1,184 participants, 140 indicated any substance use. From this sample: **Lifetime marijuana/cannabis use:** 10%.	Marijuana/cannabis was the second most commonly used illicit substance after sleeping drugs (i.e., benzodiazepines). All polysubstance users (27.1% of users) reported using marijuana/cannabis in addition to other substances.	Good
Al-Hinaai et al. (59)	Assess the prevalence of substance use and the effects of their use. A higher learning institution/university in an urban setting in Oman.	**Sample size:** n=375 **Age:** Mean 21.0 ± 2.8 **Gender:** 48.3% males	Questionnaire adapted from the Alcohol, Smoking, and Substance Involvement Screening Test (ASSIST)	**Lifetime cannabis use:** 5.1%	**Cannabis users (n=19):** -Were significantly more likely to be males (n=18) then females (n=1) (p<0.001). -Over the past 3 months, 10 never used, 6 used once/twice, and 3 used weekly. Many of the respondents who used tobacco or alcohol tried cannabis (21.6% and 35.0% respectively, p<0.001).	Good
Bajwa et al. (60)	Determine the prevalence of substance use and its contributing factors. Kuwait University and three private universities, Kuwait.	**Sample size:** n=1,473 **Age:** Mean 20.3 ± 2.9 **Gender:** 100% males	Questionnaire adapted from the World Health Organization’s guidelines “*A Methodology for Student Drug Use Surveys*”	**Lifetime Marijuana/Hashish use:** Total 11.0% Kuwait University: 6.4%; Private universities: 14.8% **Past year Marijuana/Hashish use:** Total 7.3% Kuwait University: 4.3%; Private universities: 9.7% **Past month Marijuana/Hashish use:** Total 4.4% Kuwait University: 3.1%; Private universities: 5.4%	-Cannabis (Marijuana/Hashish) was the most commonly ever used illicit substance. -Among cannabis users, 77.2% initiated use at the age of 17 or above. -Lifetime (p<0.001), past year (p<0.001), and past month (p=0.015) cannabis use were significantly higher among students attending private universities compared to those attending the public Kuwait University.	Good
Bassiony et al. (61)	Estimate the prevalence, sociodemographic and clinical correlates, and attitudes towards tramadol use. Zagazig University, Egypt.	**Sample size:** n=1,173 **Age:** Mean 20.32 ± 1.76; Range 17-34 **Gender:** 57.3% males	Drug Use Disorders Identification Test (DUDIT) and Drug Use Disorders Identification Test Extended (DUDIT-E)	**Lifetime cannabis use:** 18.8%	**The most commonly used substance was cannabis:** -Two thirds of students started with cannabis as their first substance. -At the second stage of progression in substance use, 23% used cannabis. -At the last stage, 36% used cannabis. **Among students with tramadol use:** -85.4% were using more than one drug (cannabis, alcohol, or other substances). -Cannabis use was a significant predictor of tramadol use (OR=5.30, CI 2.90-9.60, p<0.001). -Cannabis use was significantly associated with drug-related problems (Pearson correlation 20.5, p=0.015). -Cannabis use was correlated with positive thoughts about tramadol (Pearson correlation 498.0, p=0.004).	Good
Chekib et al. (62)	Determine the prevalence of lifetime illicit substance use and its risk factors. A university setting in the region of Sousse, Tunisia.	**Sample size:** n=556 **Age:** Mean 21.8 ± 2.2 **Gender:** 48.2% males	Cannabis Abuse Screening Test (CAST)	**Lifetime cannabis use:** 4.7%	Among illicit substances, cannabis was the most commonly ever used substance.	Good
Damiri et al. (63)	Describe the patterns of psychoactive substance use, knowledge, attitudes, behaviors, and associated risk factors. Four public universities in the West Bank, Palestine.	**Sample size:** n=1,383 **Age:** Range 18-≥24 **Gender:** 44.6% males	Questionnaire adapted from the European School Survey Project on Alcohol and Other Drugs (ESPAD)	**Lifetime substance use:** Natural cannabis: 4.19% Hashish: 3.90% Mr. Nice guy: 3.76% Mastaloon/Mabsatoon: 3.25% Marijuana: 2.31% Eve/Mariam: 1.51% Spice: 1.45%	Among illicit drug users, 86.7% reported being familiar with natural cannabis, hashish, or marijuana.	Good
Essadi et al. (33)	Assess the prevalence and associated factors of substance use. Mohammed First University, Morocco.	**Sample size:** n=478 **Age**: Mean 21.1 **±** 3.0; Range 18-54 **Gender:** 49.4% males	ASSIST	**Lifetime cannabis use:** 13.4% **Past 3-month cannabis use:** 9%	After tobacco and alcohol, cannabis was the most commonly ever used substance. **Based on the ASSIST score:** -25.2% of cannabis users scored 4–6 and required “*brief intervention*”. -5.6% of cannabis users scored ≥27 and required “*intensive treatment*”.	Good
Fekih-Romdhane et al. (64)	Translate and validate the Cannabis Use Intention Questionnaire (CUIQ) in the Arabic language in three countries (Egypt, Kuwait, Tunisia). Universities in Egypt, Kuwait, and Tunisia.	**Sample size:** n=2,033 Egypt: n=610 Kuwait: n=926 Tunisia: n=497 **Age:** Mean 23.25 ± 5.00 Egypt: 21.44 ± 3.01 Kuwait: 23.92 ± 6.23 Tunisia: 24.24 ± 3.61 **Gender:** 24% males Egypt: 14.6% Kuwait: 18.9% Tunisia: 45.1%	CUIQ Attitude About Cannabis Use Scale	**Lifetime cannabis use:** Total: 10.5% Egypt: 0%; Kuwait: 0%; Tunisia: 42.9% **Past six-month cannabis use:** Total: 2.3% Egypt: 100% never Kuwait: 100% never Tunisia: 91.6% never; 5.8% monthly or less; 2.2% 2–4 times a month; 0.4% 2–3 times a week	-Females scored higher than males on CUIQ scores (p<0.001). -Mean CIUQ scores were highest in Tunisia (M=34.90, SD=10.58), followed by Egypt (M=34.67, SD=6.39), and Kuwait (M=32.23, SD=7.88). -Higher CIUQ scores were significantly correlated with more favorable attitudes towards cannabis (r=0.15, p<0.001), more social dysfunction (r=0.22, p<0.001), more anxiety and depression (r=0.25, p<0.001), and more loss of confidence (r=0.08, p<0.001).	Good
Fekih-Romdhane et al. (65)	Translate and validate the Cannabis-related Psychosis Risk Literacy Scale (CPRL) in the Arabic language in three countries (Egypt, Kuwait, Tunisia). Universities in Egypt, Kuwait, and Tunisia.	**Sample size:** n=1,855 Egypt: n=558 Kuwait: n=821 Tunisia: n=476 **Age:** Mean 23.26 ± 4.96. **Gender:** 24.4% males	CPRL Attitude About Cannabis Use Scale	**Lifetime cannabis use:** 11.5% **Past six-month cannabis use:** Never: 97.7% Monthly or less: 1.6% 2–4 times a month: 0.6% 2-3 times a week: 0.1%	**-CPRL score:** 4.09 ± 1.53 **-Attitudes About Cannabis Use score:** 47.64 ± 9.41 **Factors associated with higher CPRL scores:** -Female vs. male sex (β=0.21, p=0.014). **Factors associated with lower CPRL scores:** -Kuwaiti vs. Egyptian participants (β=-1.01, p<0.001). -Rural vs. urban residency (β=-0.34, p=0.001). -Lifetime cannabis use (β=-1.27, p<0.001). -Last 6 months cannabis use (β=-1.44, p<0.001).	Good
Ghandour et al. (66)	Identify the most used prescription medications and characteristics of their users. American University of Beirut, Lebanon.	**Sample size:** n=570 **Age:** Mean 19.9 ± 0.08 **Gender:** 48.4% males	Self-developed questionnaire based on the Diagnostic and Statistical Manual of Mental Disorders IV (DSM-IV) criteria	**Lifetime marijuana use:** 19.38%	Compared to non-users, both medical and nonmedical users of prescription medications were more likely to report lifetime marijuana use: -Medical users: OR=1.78, CI 1.08-2.92, p=0.023. -Nonmedical users: OR=3.28, CI 1.84-5.83, p<0.001.	Good
Gourani (67)	Study the prevalence of drug addictions. Cadi Ayyad University, Morocco.	**Sample size:** n=418 **Age:** Mean 21.70 ± 2.46; Range 18-≥24 **Gender:** 66% males	Mini-International Psychiatric Interview, based on the DSM-IV criteria	**Lifetime cannabis use:** 9.8% **Cannabis dependence (as per DSM-IV criteria):** 75.6%	-63.4% initiated use at 15–18 years while 4.9% initiated use before 15 years of age. -70.7% initiated use with friends while 29.3% initiated use alone. -51.2% reported current regular use (34.1% on weekly basis and 17.1% on daily basis). **Lifetime cannabis use was significantly more frequent:** -In males (14.5%) compared to females (0.7%) (p=0.000). -With increasing age (18-20: 2.3%, 20-22: 7%, 22-24: 11%, ≥24: 22.3%) (p=0.000). -In those living alone (19%) or with friends (12%) compared to those living with family (4%) (p=0.040). -In those who failed ≥3 years (66.7%) compared to those who never did (3.2%), failed 1 year (14.5%), or 2 years (19%) (p=0.000). There was no difference in use based on residential area, parental salary, and faculty/year of studying at university. -35.4% of users with cannabis dependence had depression (moderate to severe) compared to 10.0% in non-dependent users (moderate only) (p=0.010).	Fair
Hamaideh et al. (68)	Assess the prevalence of types, and predictors of illicit substance use. Hashemite University, Jordan.	**Sample size:** n=835 **Age:** Range 18-28 **Gender:** 62.1% males	Self-developed questions	Of 835 participants, 145 indicated any substance use. From this sample: **Lifetime, past year, and past month use, respectively:** Hashish: 7.2%, 4.7%, 6.6% Marijuana: 1.8%, 0.8%, 0%	None.	Poor
**Hashim et al. (** 69)	Estimate the prevalence of Strox smoking. Ain Shams University, Egypt.	**Sample size:** n=558 **Age:** Not mentioned **Gender:** 75.6% males	Marijuana Smoking History Questionnaire (MSHQ)	**Current cannabis use:** 8.1% **Current Strox use:** 6.8% **Current Voodoo use:** 0.5%	-Males were more likely to smoke cannabis (p=0.001) and Strox (p=0.004) than females. -There was a significant association between tobacco and Strox consumption (p<0.001) and between cannabis and Strox consumption (p<0.001). Strox users were likely to be smokers of tobacco and cannabis. **Reasons for smoking Strox:** -Achieving euphoria (28.9%) -Treating depression (23.7%) -Experimentation (23.7%) -Peer pressure (21.1%) -Having excess money (2.6%)	Fair
Helou et al. (70)	Determine whether a relationship exists between quality of life and substance use among medical students. Medical schools of private and public universities in Lebanon.	**Sample size:** n=465 **Age:** Mean 22.06 ± 1.67 **Gender:** 54% males	Revised Cannabis Use Disorders Identification Test (CUDIT-R)	**Past 6-month cannabis use:** 16.1% **Cannabis use disorder:** 4.1%	Higher cannabis use disorder scores were significantly associated with higher physical Quality of Life scores (p<0.001).	Good
Jebali et al. (71)	Determine the prevalence, dependence, and associated factors of psychoactive substance use. Private nursing institute in Tunisia.	**Sample size:** n=455 **Age:** Mean 22.8 ± 2.6 **Gender:** 51.4% males	CAST	**Past year cannabis use (with or without another substance):** 20.2% **Past year cannabis use (alone):** 0.2%	95.7% of participants scored high on the risk for cannabis dependence. In univariate analysis, cannabis dependence was significantly associated with male gender (p=0.001) and living away from family (p<0.001) (lost significance in multivariate analysis).	Good
Kabbash et al. (72)	Identify the prevalence of tobacco, alcohol and drug use and associated factors. Kafr El-Sheikh University, Egypt.	**Sample size:** n=2,252 **Age:** Mean 20.03 ± 1.3; Range 17-25 **Gender:** 47.3% males	Questionnaire designed by the Fund for Drug Control and Treatment of Addiction (FDCTA) in Egypt	**Lifetime hashish use:** Total 6.6% Males 13.5%; Females 0.3% **Past month hashish use:** Total 3.6% Males 7.6%; Females 0% **Past month Bhang use:** Total 1.4% Males 2.9%; Females 0%	After tobacco and alcohol, hashish was the most commonly ever used substance. -Lifetime (p<0.001) and past month (p<0.001) use of Hashish and Bhang were significantly more frequent among male than female students. -Hashish use was significantly more frequent among students in practical faculties (4.4%) than those in theoretical (4.0%) and medical (1.4%) faculties (p=0.013). -Attitude toward self-initiation of Hashish/Bhang: 73.9% impossible, 12.6% very difficult, 4.8% difficult, 4.2% easy, 2.3% very easy, and 2.2% don’t know. -Strong disagreement towards trial, occasional, or regular use of Hashish/Bhang: 64.8%, 65.1%, and 70.2%, respectively. -Perception of smoking 1–2 times only, occasionally, or regularly Hashish/Bhang as high danger: 64.7%, 67.5%, and 83.5% respectively.	Fair
Kabbash et al. (73)	Identify the prevalence and associated determinants of drug use. Tanta University, Egypt.	**Sample size:** n=7,445 **Age:** Mean 20.56 ± 1.33 **Gender:** 36.7% males	Self-developed questions	**Lifetime substance use:** **Hashish:** Total 4.5% Males 4.6%, Females 4.5% **Bhang:** Total 1.6% Males 1.7%, Females 1.5% **Synthetic cannabinoids:** Total 0.4% Males 0.5%, Females 0.4% **Current use:** **Hashish:** Total 1.9% Males 2%, Females 1.8% **Bhang:** Total 0.5% Males 0.6%, Females 0.5% **Synthetic cannabinoids:** Total 0.2% Males 0.3%, Females 0.1%	There was no significant different between males and female in the lifetime prevalence or current use of Hashish, Bhang, and synthetic cannabinoids. -Students in theoretical faculties had a higher prevalence of Hashish use (3.5%) than those in practical (1.3%) and medical (0.2%) faculties (p<0.001). -Students in theoretical faculties had a higher prevalence of Bhang use (1.1%) than those in practical (0.2%) and medical (0.1%) faculties (p<0.001). -Students in theoretical faculties had a higher prevalence of synthetic cannabinoids use (0.4%) than those in practical (0.03%) and medical (0.1%) faculties (p<0.01).	Fair
Karam et al. (74)	Determine the prevalence of substance use and associated risk factors. American University of Beirut and Saint Joseph University, Lebanon.	**Sample size:** n=1,851 **Age:** Range 16-≥22 **Gender:** 51.5% males	Questionnaire adapted from Diagnostic Interview Schedule (DIS) version 3	**Lifetime cannabis use:** Total: 2.6% Males 3.7%; Females 0.7% 16–18 years: 2.4%; 19–21 years: 2.5%; ≥22 years: 2.6%	-Of all illicit drugs, cannabis had the highest rate of admitted abuse (0.3%). -0.2% of cannabis users reported dependence. -Males were significantly more likely to ever use cannabis than females (p=0.000). -There was no significant difference in cannabis use between age groups.	Fair
**Karam et al. (38)	Describe the extent and patterns of alcohol and illicit substances use. Ten private schools and two of the largest private universities in Lebanon.	**University sample:** **Sample size:** n=1,837 **Age:** 20.2 ± 1.6 **Gender:** 47.4% males	Diagnostic Interview Schedule version IV, based on the DSM-IV criteria	**Lifetime Hashish/Marijuana use in university sample:** Total 8.8% Males 11.4%; Females 6.3%	**Among the university sample:** - Lifetime cannabis use was significantly higher in males compared to females (p<0.005). -Age of onset of ever using cannabis: 17.7 ± 2.6 -15.6% were for the legalization of hashish/marijuana.	Good
Khafagy et al. (75)	Detect the prevalence and associates of substance use. Mansoura University, Egypt.	**Sample size:** n=1,138 **Age:** Mean 19.6 ± 1.4; Range 17-25 **Gender:** 57.1% males	DUDIT	Lifetime illicit substance use: 6.5% Among substance users, 24.3% used cannabis.	Among illicit substances, cannabis was the most commonly ever used substance. 35.1% were polysubstance users of whom 13.5%, 16.2%, 4.1%, and 1.4% used cannabis and tramadol; cannabis and alcohol; cannabis and inhalant; and cannabis, alcohol, and tramadol, respectively.	Good
Khedr et al. (76)	Assess the prevalence of psychoactive substance use. Aswan university, Egypt.	**Sample size:** n=1,440 **Age:** Range 16->19 **Gender:** 71.9% males	Substance Misuse Questionnaire of Egypt Drug Abuse Screening Test 20 (DAST-20)	*Hashish (17.59%) was the most commonly used substance after nicotine.	None.	Fair
Kjiri et al. (77)	Compare the prevalence of substance use among males and females. Three universities in Morocco.	**Sample size:** n=1,208 **Age:** Not mentioned **Gender:** 34.8% males	Self-developed questions	*Cannabis use: 11.7%	Cannabis use was significantly more common in males compared to females (p<0.001). **Excluding tobacco, cannabis was the:** -second most commonly used substance in males (following alcohol). -third most commonly used substance in females (following alcohol and benzodiazepines).	Fair
Mina et al. (78)	Create and validate two scales in the Arabic Language to assess perceived ha towards cannabis use and attitude against cannabis use. The Lebanese University, Lebanon.	**Sample size:** n=415 **Age:** Mean 20.96 ± 2.67 **Gender:** 41.2% males	Self-developed questions	**Lifetime cannabis use:** Recreational purposes: 32% Medicinal purposes: 7.5% Recreational and medicinal purposes: 6.7%	**Perceived knowledge about cannabis use scale score:** 71.49 ± 8.44 (median=73) **Attitudes about cannabis use scale score:** 49.42 ± 3.87 (median=50) **Factor associated with higher perceived knowledge about cannabis:** -Male vs. female sex (p=0.012). -Cannabis use for recreational purposes (p<0.001). -Stronger attitude about cannabis use (r=0.189, p<0.001). -Older age (r=0.105, p=0.033). **Factor associated with greater mean attitude about cannabis use:** -Male vs. female sex (p=0.007). -Cannabis use for recreational purposes (p=0.002). -Cannabis use for medicinal purposes (p=0.037).	Fair
Moaouad et al. (79)	Compare the prevalence of substance use between medical and non-medical students. Saint Joseph University, Lebanon.	**Sample size:** n=280 **Age:** Range 17-24 **Gender:** 49.29% and 45% males from medical and non-medical faculties, respectively	Mini-International Psychiatric Interview, based on the DSM-IV criteria	**Cannabis dependence (as per DSM-IV criteria):** -Males: 1.45% vs. Females: 0% -Medical students: 0.71% vs. Non-medical students: 0%	No significant difference in cannabis dependence was found between gender or student groups.	Poor
Naguib et al. (80)	Estimate the prevalence of cannabinoid abuse and explore risk factors. A randomly chosen university in Egypt.	**Sample size:** n=2,380 **Age:** Mean 20.5 ± 1.35; Range 18-24 **Gender:** 66.1% males	Addiction Severity Index (5^th^ Edition)	*Illicit/cannabinoid substance use: 4.9% Among substance users, the most used substances were Hashish (96.5%), Strox (41.3%), Bhang (34.4%), and Voodoo (34.4%). The illicit drugs that students reported using regularly were Strox (33.3%), Bhang (30%), and Voodoo (30%).	-Most users were males (93.1%) and in practical college (79.3%). -About half were living in rural areas (51.7%) and had low (50.8%) or moderate (41.3%) socioeconomic status. *Factors associated with cannabinoid use: - -Male gender: OR=5.46, CI 2.40-12.44, p=0.001. - -Family history of conflict: OR=6.48, CI 5.08-8.64, p<0.001. - -Family history of substance use: OR=1.98, CI 1.48-2.08, p=0.045. - -History of child abuse: OR=2.85, CI 1.96-3.04, p=0.001. - -Encouragement by peers: OR=2.95, CI 1.73-5.05, p<0.001. - -Having a stay-at-home mother: OR=1.56, CI 1.19-2.04, p=0.001. - -Living in an urban area: OR=2.22, CI 1.53-5.00, p=0.002.	Fair
Osman et al. (81)	Assess the magnitude, circumstances, and factors associated with substance use. Private university in Khartoum, Sudan.	**Sample size:** n=410 **Age:** Mean 19.6 ± 1.9 **Gender:** 29.8% males	Questionnaire adapted from the World Health Organization student drug surveys	**Lifetime cannabis use:** 9.3% **Past year cannabis use:** 7.1% **Past month cannabis use:** 4.9%	The prevalence of cannabis use (lifetime, past year, and past month) was the highest after tobacco. Most students initiated cannabis use at the age of 17 and above (no percentage reported).	Fair
Salameh et al. (82)	Assess the prevalence, determinants, and risk factors of substance use. The Lebanese University and several private universities in Lebanon.	**Sample size:** n=1,945 **Age:** Range 17-≥21 **Gender:** 46.2% males	ASSIST	**Lifetime cannabis use:** Total 12.3% Lebanese university: 9.6%; Private universities: 14.5% **ASSIST scores among users:** **Lebanese university:** 6.1% scored 0-3 (no intervention) 2.9% scored 4-26 (brief intervention) 0.6% scored >27 (intensive intervention) **Private universities:** 8.9% scored 0-3 (no intervention) 5.4% scored 4-26 (brief intervention) 0.2% scored >27 (intensive intervention)	-The most commonly used substance was cannabis. -Over the past 3 months, 1.1%, 0.9%, and 0.5% of cannabis users reported monthly, weekly, and daily use, respectively. -There was no significant difference in lifetime cannabis use or ASSIST scores between the Lebanese University and private universities. **Lifetime cannabis use was significantly more frequent:** -In males (16.9%) compared to females (8.5%) (p<0.001). -With increasing age (17-18: 8.9%, 19-21: 10.2%, >21: 20.3%) (p<0.001). -A good relationship with parents was protective towards cannabis use (OR=0.40, CI 0.18-0.86, p=0.024). -A higher number of cigarettes consumed per day (OR=1.65, CI 1.35-2.01, p<0.001) or narghiles per week (OR=1.36, CI 1.09-1.69, p=0.006) was associated with increased cannabis use.	Good
Talih et al. (83)	Evaluate the prevalence of burnout, mood and anxiety symptoms, and substance use. Medical school at the American University of Beirut, Lebanon.	**Sample size:** n=172 **Age:** Range 20->25 **Gender:** 51.16% males	Drug Abuse Screening Test 10 (DAST-10)	**Lifetime cannabis use:** 31.9%	-Among illicit substances, cannabis was the most commonly ever used substance. -There was a significant difference in cannabis use among the four medical school years. First year medical students had lower rates of cannabis use than second-, third-, and fourth-year students (χ2 = 12.93, p=0.005). -Cannabis use was not significantly associated with burnout.	Fair

*The type of measured prevalence (i.e., lifetime, past year, etc.) is not specified; **this study is also displayed in **Table 1**.

### 3.1 Studies exploring cannabis use among students attending a school setting

#### 3.1.1 Characteristics of the included studies

A total of 20 studies explored the characteristics of cannabis use among students in school settings. Data emanated from eleven Arab countries: Palestine (n=6) (41–45, 50), Egypt (n=4) (47, 53–55), Morocco (n=4) (46, 50, 52, 57), Algeria (n=2) (50, 52), Iraq (n=2) (48, 51), Kuwait (n=2) (49, 51), Jordan (n=1) (40), Lebanon (n=1) (38), Mauritania (n=1) (52), Saudi Arabia (n=1) (39), and Tunisia (n=1) (56) (**Table 1**).

In terms of focus, 5 studies directly assessed cannabis use as a primary outcome (40, 50–52, 57) while the remaining studies had broader aims, addressing substance use in general, including cannabis.

#### 3.1.2 Descriptive statistics

Sample sizes ranged from 140 (54) to 10,648 participants (53). Participants were generally school-aged, but some studies included students older than 18. Specifically, 3 studies (39, 48, 55) included participants with an age range reaching 19. The study with the lowest mean age was conducted by Zammit et al. in Tunisia, reporting a mean age of 13.3 years (56), while Soliman et al. had the highest mean age of 17.27 years (55). The proportion of male participants ranged widely across studies, from 31.7% (53) to 100% (39, 55). Most studies focused on students attending secondary schools (grades 7-12), but some targeted younger students (grades 6-10) or specific groups such as vocational school students (55).

#### 3.1.3 Instruments used to assess cannabis use

The most commonly used instrument was the Global School-Based Student Health Survey (GSHS), utilized in 4 studies (50–53). Other instruments included the Cannabis Abuse Screening Test (CAST) (40), the Diagnostic Interview Schedule version IV (38), and questionnaires adapted from different sources as follows: the European School Survey Project on Alcohol and Other Drugs (ESPAD) (44, 46), the National Epidemiologic Survey on Alcohol and Related Conditions (NESARC) (45), the United Nations Office on Drugs and Crime (UNODC) (48), the 144-item 1998 New Jersey Triennial Public High School Survey of Drug and Alcohol Use questionnaire (49), the Addiction Severity Index Scale (53), and the World Health Organization (WHO) (54). Seven studies used self-developed instruments, tailored to local contexts and cultural sensitivities.

#### 3.1.4 Prevalence of cannabis use

Studies used various prevalence measurements and terminologies to describe cannabis use. Some studies categorized cannabis, hashish, and marijuana separately, while others grouped them together. Accordingly, the results showed wide variations. Lifetime use of cannabis/hashish/marijuana ranged from 0.7% in Iraq (48) to 9.4% in Morocco (46). Past-year use ranged between 0.3% in Iraq (48) and 18% in Palestine (45). Past-month use displayed a broader range, from 0.3% (48) to 24% (55).

In addition, several studies examined synthetic cannabinoids. Damiri and colleagues explored a wide range of cannabinoids such as Spice and Mr. Nice Guy, reporting a combined lifetime prevalence of 12.8% (44). Shaheen et al. examined the use of Bango, Strox, and Voodo (54) while Loffredo and colleagues assessed Bango use (47).

Five studies reported cannabis as the most commonly used illicit substance among students (39, 44, 55–57). However, Rabie and colleagues found cannabis to be the second most common, after organic solvents (not formally defined, but generally referring to inhaled substances such as glue, paint thinner, or correction fluid) (53).

#### 3.1.5 Significant correlates of cannabis use

##### 3.1.5.1 Gender

Male students consistently reported higher cannabis use than females across all studies discussing gender differences. For instance, Zarrouq et al. reported 13.5% lifetime cannabis use in males versus 1.9% in females (57), and El Omari et al. showed similar gender differences (males 9.5% vs. females 2.1%) (46). This pattern held across countries, with statistical significance (p<0.05) reported in several studies (38, 40–43, 45, 47, 51).

##### 3.1.5.2 Age

The age of initiation of cannabis use varied between 15 (46) and 16 (38). Older students tended to report higher cannabis use. Loffredo et al. found lifetime hashish use to be significantly lower in 12-14- and 14–16-year-old individuals, compared to the group >16 years (47). Alternatively, in Diamond et al., although past-year cannabis use was highest among 15-16-year-olds (21.9%), both those below 14 and above 17 reported less past-year use (19% and 8.5%, respectively) (45).

##### 3.1.5.3 Family, religion, and sociocultural factors

Family and social dynamics were identified as important predictors. Cannabis use was more common among students with family problems and those not living with both parents (40–42), among students sleeping outside home (46) or not living with parents (47), and among those with family member/guardian/friend who consumed alcohol (46) or tobacco (51).

Religion was another significant factor, with lower levels of religiosity (i.e., low vs. high; secular vs. devout) linked to higher cannabis use in multiple studies (41–43, 45). Peltzer and Pengpid also noted higher cannabis use in students from socioeconomically disadvantaged backgrounds, specifically those living with food insecurity (51).

Beyond family and sociocultural factors, some studies explored the role of academic performance. El Omari and colleagues found that students absent from school or with lower grades were more likely to use cannabis (46), while Loffredo et al. and Soliman et al. linked a positive work status and working at night, respectively, with higher use (47, 55).

##### 3.1.5.4 Mental health correlates

Peltzer & Pengpid found that students with current tobacco use, anxiety, or suicidal ideation were more likely to use cannabis (51). Similarly, Alzyoud found a positive relationship between cannabis use and having a high-risk mental status problem (40).

##### 3.1.5.5 Attitudes, Beliefs, and Perceptions

Most students had good knowledge about cannabis. Alzyoud found that about three-quarters (77.1%) had heard about cannabis (40). Damiri et al. noted most students (74.7–97.3%) had heard about the substance (44) and 91% of participants in El Omari’s study reported good knowledge about hashish and related products (46). Alzyoud noted that a majority viewed cannabis use as a problem (69.8%), against society norms (80.0%), and problematic during adolescence (81.3%) (40). Along the same lines, Karam et al. noted that 57.3% of participants thought that the use of cannabis was a crime. Some studies highlighted the influence of attitudes on cannabis use (38). According to Alzyoud, students having positive attitudes towards use and the belief that cannabis consumption helps in forming friendships were significantly more likely to use it (40). El Omari and colleagues also found that students who perceived cannabis as harmless were more likely to report usage (46).

### 3.2 Studies exploring cannabis use among students attending a university setting

#### 3.2.1 Characteristics of the included studies

A total of 29 studies explored the characteristics of cannabis use among students in university settings. Data emanated from nine Arab countries: Egypt (n=9) (61, 64, 65, 69, 72, 73, 75, 76, 80), Lebanon (n=8) (38, 66, 70, 74, 78, 79, 82, 83), Morocco (n=4) (33, 58, 67, 77), Tunisia (n=4) (62, 64, 65, 71), Kuwait (n=3) (60, 64, 65), Jordan (n=2) (32, 68), Palestine (n=1) (63), Oman (n=1) (59), and Sudan (n=1) (81).

In terms of focus, 5 studies directly assessed cannabis use as a primary outcome (64, 65, 69, 78, 80), while the remaining studies had broader aims. These studies often explored the prevalence, patterns, and correlates of various substances, with cannabis emerging as one of the most commonly discussed illicit drugs.

#### 3.2.2 Descriptive statistics

Sample sizes ranged from 172 (83) to 7,445 participants (73). The age range of participants varied between 16 and 28 years, while the mean age ranged between 19.6 years (75, 81) and 24.24 years (64). The proportion of male participants ranged widely across studies, from 14.6% (64) to 100% (60). Few studies particularly focused on medical (70, 83) and nursing (71) students.

#### 3.2.3 Instruments used to assess cannabis use

The most frequently used instrument was the WHO-based Alcohol, Smoking, and Substance Involvement Screening Test (ASSIST), employed in 3 studies (33, 59, 82). Three other studies also used instruments derived from the WHO (32, 60, 81). The CAST was used in two studies (62, 71), as well as the Drug Use Disorders Identification Test (DUDIT) (61, 75). Two studies used a questionnaire based on the Mini-International Psychiatric Interview (67, 79). The other studies used different established or self-developed questionnaires, as detailed in **Table 2**.

#### 3.2.4 Prevalence of cannabis use

As with studies tackling schools, studies in university settings used various prevalence measurements and terminologies to describe cannabis use. Although results showed wide variations, prevalence numbers were consistently higher compared to school settings.

Lifetime use of cannabis ranged from 4.7% in Tunisia (62) to 32% in Lebanon (83) and Egypt (78). Lifetime hashish and marijuana use reached a peak of 7.2% in Jordan (68) and 19.38% in Lebanon (66), respectively. Past-year cannabis use reached 20.2% in Tunisia (71). Past-month cannabis/hashish/marijuana use displayed a broader range, from 0% for marijuana (68) to 6.5% for hashish (68).

Several studies examined synthetic cannabinoids. Damiri and colleagues explored a wide range of cannabinoids such as Mr. Nice Guy, Mastaloon/Mabsatoon, and Eve/Mariam (63). Although Eve is commonly used as a street name for 3,4-methylenedioxymethamphetamine (MDMA) or ecstasy, it was classified as a synthetic cannabinoid in the study. Hashim et al. (69) reported the current use of Strox to be 6.8%. Kabbash et al. found past month Bhang use of 1.4% (72). In a more recent study, the same team noted a lifetime prevalence of Bhang and synthetic cannabinoids of 1.6 and 0.4%, respectively (73). Naguib et al. highlighted that the illicit drugs regularly used were Strox (33.3%), Bhang (30%), and Voodoo (30%) (80).

Twelve studies reported cannabis/hashish as the most commonly used illicit substance (33, 58, 60–62, 72, 74, 75, 80–83). Only Alsammak and colleagues found cannabis to be the second most common, after sleeping drugs (not formally defined, but mentioned to include benzodiazepines) (32), and Kjiri et al. had similar findings among female users (77).

Cannabis dependence significantly varied from 0.2% (74) to 75.6% (67), while the percentage of cannabis users requiring intensive intervention as per ASSIST scores reached 5.6% (33).

#### 3.2.5 Significant correlates of cannabis use

##### 3.2.5.1 Gender

Consistently, male students reported higher cannabis use than their female counterparts (38, 59, 67, 69, 72, 74, 77, 80, 82). Only Kabbash et al. and Moaouad et al. did not find a significant gender difference in cannabis use (73, 79). Fekih-Romdhane and colleagues found females to score significantly higher than males on the Cannabis Use Intention Questionnaire (64).

##### 3.2.5.2 Age

Older students tended to report higher cannabis use. Gourani found that lifetime cannabis use significantly increased with age, with those aged 22–24 reporting a rate of 22.3% compared to 2.3% among those aged 18-20 (67). Similar significant findings were discussed by Salameh and colleagues, with 20.3% versus 8.9% rates among the age groups >21 and 17-18, respectively (82).

Three studies highlighted that most users started their cannabis use around the age of 17 (38, 60, 67), and two others noted cannabis as the most common first illicit substance to be tried by students (58, 61).

##### 3.2.5.3 Family and sociocultural factors

Cannabis use was significantly more common in those living alone or with friends compared to those living with families (67), those with a family history of conflict, those with a family history of substance use, and those encouraged by peers (80). Along the same lines, a good relationship with parents was significantly protective towards cannabis use (82).

Other identified correlates included academic performance, where lower achievement was significantly associated with higher cannabis use (67). Additionally, Bajwa et al. found cannabis use to be significantly more common among those attending private versus public universities (60). Talih and colleagues also noted significantly increasing rates of cannabis use throughout the four medical school years (83).

##### 3.2.5.4 Mental health correlates

Beyond demographic and social factors, substance co-dependence and mental health conditions were frequently associated with cannabis use. For instance, a higher consumption of tobacco or narghile (i.e., shisha) was found to be significantly associated with increased cannabis use (82). Bassiony et al. found cannabis use to be a significant predictor of tramadol use (61). Ghandour et al. noted that, compared to non-users, both medical and nonmedical users of prescription medications were more likely to report lifetime marijuana use (66). Those with cannabis dependence were significantly more likely to have depression than non-dependent users (67). Lastly, a history of childhood abuse was significantly associated with cannabinoid use (80).

##### 3.2.5.5 Attitudes, beliefs, and perceptions

Damiri and colleagues noted a high level of familiarity with cannabis and related products among most (86.7%) illicit drug users (63). Recreational use was significantly associated with higher perceived knowledge and attitude scores about cannabis use (78). In parallel, more favorable attitudes toward cannabis were associated with higher usage scores (64). One study found mostly negative attitudes toward self-initiation of Hashish/Bang and strong disagreement towards their use (72). Hashim et al. noted achieving euphoria (28.9%), treating depression (23.7%), and experimentation (23.7%) as the most common reasons to use Strox (69).

## 4 Discussion

This systematic review offers valuable insights into the phenomenon of cannabis use among school and university students in the Arab World. The 48 analyzed studies spanned many Arab nations, primarily emanating from Egypt, Lebanon, Morocco, and Palestine. Most studies provided information on the prevalence of cannabinoid use, characteristics of users, and correlates of consumption. Sample sizes ranged from small groups of less than 200 students to large-scale cross-sectional designs involving over 5,000 participants. The mean age of students ranged from 13 to 24 years, with a male-dominated demographic in most studies. A variety of instruments were used to assess cannabis use including standardized questionnaires, such as ASSIST, and self-developed customized local surveys.

The prevalence of cannabis use varied widely across studies, ranging from less than 2% to over 30%, depending on the methodology, type of prevalence measured, and the specific cannabinoid considered. This variation also reflects differences in social and cultural attitudes toward substance use, reporting behaviors, and legal frameworks across Arab nations. Regardless, most studies identified cannabis as the most commonly used illicit substance among school and university students. Significant correlates of use included gender, with males more likely reporting use than females; age, with older students displaying higher usage rates; and attitudes, with more liberal views toward drug use being associated with higher consumption. Other relevant predictors included family dynamics, peer influence, religiosity, and academic performance.

Overall, these findings indicate a complex interplay of sociocultural and geographical factors that influence cannabis use among students in the Arab World. The results highlight potential avenues for future intervention strategies and policy formulations.

### 4.1 Cannabis use across school and university settings

Although cannabis consumption was noted to be a significant issue across both school and university settings, the prevalence of use was higher in the latter group. This was evidenced by lifetime use reaching as high as 32% among university students (78, 83), compared to 9.4% in schools (46). This variation may be attributed to various factors, including older age. Indeed, in the included studies, age was found to be a significant correlate of cannabis use, with older students consistently displaying higher rates of cannabis use compared to their younger counterparts (45, 47, 67, 82). This finding echoes international data. For instance, an 8-year longitudinal Canadian study noted older age as one risk factor for daily cannabis use in younger adults (84). Along the same lines, in 2023, marijuana use among individuals aged 18 and older in the USA was reported at 50.5% for lifetime use, 22.9% for past year use, and 16.3% for past month use. In contrast, among those aged 12 to 17, the rates were significantly lower, with 13.4% reporting lifetime use, 11.2% past year use, and 6% past month use (85).

Other factors contributing to this variation in results between school and university settings include greater peer influence at the university (86, 87), increased accessibility of cannabis, especially in the context of legislative changes and higher exposure to more permissive social norms (88, 89) compared to the closely-knit school environments, and potentially greater independence (90).

These findings highlight that the transitional years between school and university constitute a crucial point of intervention during which students might be at increased risk of initiating or increasing cannabis use. Cannabis use should be systematically assessed during this period, particularly among high-risk individuals.

### 4.2 Cannabis use across Arab countries

Differences in the rates of cannabis use were evident between Arab nations. Some countries, such as Egypt, Lebanon, and Morocco consistently reported some of the highest prevalence rates in both school and university settings. In Egypt, cannabis farming is partially authorized (91). Lebanon and Morocco are known for their historical ties to cannabis cultivation and have been key exporters of hashish for the past five decades. In Lebanon’s Beqaa Valley and Morocco’s Rif region, cannabis cultivation has represented a lifeline for disadvantaged farmers, driven by local, regional, and international demand. The suitable climate, marginalized economic status of these areas, and global prohibition efforts have paradoxically sustained cannabis cultivation as a dynamic part of these countries’ economies (28). In Morocco’s competitive cannabis industry, various regions specialize in different forms of hashish. The stability of the market contrasts with Lebanon, where geopolitical instability has resulted in a disorganized market supply (28). However, Lebanon made a significant shift in 2020 by legalizing cannabis for medical and industrial purposes, becoming the first Arab nation to do so (92). This legalization could potentially increase recreational use, particularly among the youth population.

On the other hand, studies from countries such as Iraq and Saudi Arabia noted substantially lower rates of cannabis use. This can be attributed to religious factors, as Islam’s strict prohibition against the use of intoxicating substances, including cannabis, can contribute to lower prevalence rates. In conservative societies that portray drugs as morally corrupting, legal frameworks align with religious doctrine by implementing strict penalties for substance use, which acts as a deterrent for individuals from engaging in cannabis use (93–95). In that regard, although religious and legal prohibitions might suppress cannabis use, the reported rates are likely underestimated due to individuals’ reluctance to openly disclose consumption for fear of repercussions. Furthermore, as globalization progresses, even nations with strict bans, such as Saudi Arabia, are increasingly exposed to Western cultures that depict drug use as socially accepted behavior (96). This has raised concerns about potential increases in cannabis and drug consumption, particularly among vulnerable youth, although several reviews from the US and Europe highlighted mixed effects on the prevalence of cannabis use and its effects among youth and adolescents following legalization and policy changes (97, 98). This is modulated by several interrelated factors, including laws and the extent of their application, socioeconomic status, and sociocultural background (98).

Interestingly, several studies analyzed the prevalence of region-specific cannabinoid products and referenced local slang nomenclature. These included Bhang/Bango, informal terms referring to natural cannabis. In Egypt, other commonly reported names refer to synthetic cannabinoid products, such as Voodoo and Strox. The latter is currently the most popular synthetic cannabinoid in Egypt and is typically composed of dried plant material (sometimes including toxic plants such as Atropa belladonna, Datura, or Hyoscyamus), sprayed with unidentified synthetic cannabinoids (99). In Palestine, other local nomenclatures were reported, including Eve/Mariam, Hydro, Mastalon, Mabsoton, and Mr. Nice Guy. While no published studies have specifically analyzed the composition of these substances, Mr. Nice Guy is widely recognized in the market as a synthetic cannabinoid product. The remaining nomenclatures are also believed to refer to synthetic cannabinoids, although their exact composition remains undocumented and requires further investigation.

The cultural acceptance of cannabis in historically significant regions such as Lebanon and Morocco, combined with globalization and evolving international policies – including the decriminalization and legalization of cannabis for medical or recreational use, may contribute to increased cannabis use among youth in the Arab World. These findings underscore the need for culturally sensitive intervention strategies that account for not only the sociocultural, religious, and legal contexts of each Arab nation, but also the evolving global trends and perceptions towards cannabis.

### 4.3 Prevalence of cannabis use

Lifetime prevalence rates of cannabis use in the Arab World ranged from 0.7% (48) to 32% (78, 83), indicating significant variability across countries, which can be attributed to the abovementioned factors. Most results clustered around the range of 5-15%. In comparison, marijuana use in the US in 2023 was reported to be much higher, with 50.5% of individuals aged 18 and above reporting lifetime use. In contrast, among those aged 12 to 17, the rate is more comparable at 13.4% (85). A systematic review of the prevalence of cannabis vaping among adolescents in the US and Canada in 2019–2020 identified a lifetime pooled prevalence of 13.6% (100). In sub-Saharan Africa, a meta-analysis of 53 studies noted a lifetime prevalence of cannabis use among adolescents to be 7.9% (101). A systematic review focusing on medical students in India identified a pooled prevalence of cannabis use of 8.2% (102). These differences highlight the importance of considering socio-cultural and legal contexts when interpreting cannabis use data.

In the included studies, cannabis was often found the most commonly used illicit substance among school and university students, a trend observed globally as well. For instance, according to the Monitoring the Future Survey, cannabis has remained the most frequently used illicit substance among school students in the US over at least the past decade (103). Similarly, the latest European statistics indicate that cannabis is the most commonly consumed illicit drug among young adults (104). Among specific subgroups, such as medical students, cannabis also ranks as the most frequently used illicit substance (105).

Additionally, findings from the analyzed studies noted that early initiation of cannabis use was a critical factor in continued use into later years. Compared to late initiators, early cannabis users tend to have more externalizing mental health symptoms, a history of trauma, and concerns about violence (106). Predictors of early adolescent onset cannabis use include anxiety symptoms (107), cigarette use, and drinking to the point of intoxication (108). In their comprehensive review, Padoan and colleagues raise concerns about pediatric cannabis use. They highlight short-term effects including social isolation, mood changes, and suicidal tendencies, as well as long-term consequences, such as neurocognitive impairment, psychiatric disorders, respiratory issues, cardiovascular complications, and immunotoxicity (109).

Given these risks, special attention should be given to students at increased risk for early cannabis use, including those with exposure to other substances or a predisposition to mental health conditions. Proactive preventive measures and early intervention programs should be tailored to address the needs of this vulnerable population, particularly in regions with shifting legal and sociocultural landscapes.

### 4.4 Gender, family dynamics, and sociocultural factors

Consistent with previous research (110, 111), the present review reaffirms that being male is a significant predictor of cannabis use. Sex-based differences in the endocannabinoid system have been documented (110), while gender-related disparities may be influenced by societal norms, with substance use being more socially accepted and permissible for boys and men than for girls and women (111). This discrepancy has major repercussions, as males consistently report more cannabis-related negative consequences than females (112).

Family dynamics, specifically parental conflict or not living with parents, emerged as a key influence on cannabis use. Peer pressure was also found to be a significant factor associated with cannabis use. These findings echo international research. For instance, an 8-year longitudinal investigation in Canada identified higher levels of family stress, among other risk factors, as significantly increasing the odds of daily cannabis use among young adults (84). In another Canadian study, among 14- to 18-year-olds, higher levels of parental monitoring were associated with lower odds of cannabis use, while having peers who smoked cigarettes increased the odds (113). The importance of peer influence on adolescent cannabis use has been established in a substantial body of research worldwide (114, 115). One systematic review identified friends’ cannabis use as the strongest predictor of personal cannabis use. Adolescents were also more likely to consume cannabis if they felt disconnected from school peers and had cannabis-using friends, especially at younger ages (87). One study in the US highlighted that first-year university students were more likely to select peers with similar past-month marijuana use. Furthermore, a student’s past-month marijuana use became more similar to their peers’ use over time (86).

Sociocultural factors, such as lower religiosity, poor academic performance, and disadvantaged socioeconomic status, also emerged as predictors of cannabis use. Research has established that religion moderates cannabis use (116), with higher religiosity being associated with lower use (117, 118). Early-age cannabis use increases the probability of poor cognitive function, and persistent consumption can be a barrier to academic achievement (119, 120). Lastly, consumption has been consistently higher among adolescents with the most socioeconomic adversities (121, 122).

These findings highlight the complex influences on adolescent and young adult cannabis use, including male gender, family dynamics, peer pressure, and sociocultural factors. Interventions should focus on strengthening family relationships, promoting peer resistance skills, and providing support for disadvantaged youth. Additionally, school- and college-based programs should educate students about the risks of early cannabis use, fostering a supportive environment to mitigate these risks effectively.

### 4.5 Mental health and cannabis use

The role of mental health was explored in several studies, revealing an association between cannabis use and psychiatric manifestations such as anxiety (51, 64), depression (64, 67), and suicidal ideations (51). These findings align with a broader body of evidence linking cannabis use in youth to various mental health conditions, including mood (123, 124) and anxiety disorders (125, 126), as well as increased risk of suicidality (127, 128). Importantly, cannabis use has also been associated with an increased risk of psychosis and schizophrenia, particularly among individuals with a genetic or familial vulnerability. However, this relationship is complex and may be moderated by factors such as age of initiation, frequency of use, and cannabis potency (129, 130). A recent scoping review by Baral et al. further emphasized the mental health consequences of cannabis use among young individuals (131), reinforcing the need for greater awareness among students, families, and healthcare providers.

Beyond mental health, a substantial body of research underscores the broader consequences of early and sustained cannabis use. Evidence indicates negative outcomes including impaired cognitive functioning, reduced physical health, and compromised overall well-being (132). Cannabis use is also associated with poorer academic outcomes, including decreased school performance, lower academic achievement, and higher rates of absenteeism (133). In the long term, chronic use has been linked to a trajectory of social and economic disadvantage, including increased likelihood of being poor, unmarried, underemployed, and experiencing anxiety (134). Moreover, growing evidence implicates cannabis use as a potential contributing factor to the onset or worsening of psychiatric disorders, particularly among adolescents and individuals at elevated risk (135, 136).

Taken together, these findings highlight the urgent need for proactive, evidence-informed public health responses. In this context, the current systematic review carries important implications for policy, practice, and future research concerning cannabis use among students in the Arab World.

The high prevalence of use among university students highlights the pressing need for early, school-based, family, and community intervention strategies, ideally beginning in secondary school. These should include age-appropriate, evidence-based prevention programs targeting high-risk individuals—particularly male students facing family difficulties, mental health challenges, and peer pressure. Effective programs should adopt a comprehensive approach that includes anti-drug education, refusal skills, social-skills training, and self-management strategies (137), while also fostering resilience and promoting positive youth development (138). Embedding family interventions within school settings through proactive parental engagement is also recommended (139), particularly in the Arab World, where collectivist norms prevail. In this context, strategies should be culturally adapted and gender-sensitive to enhance their relevance and impact. A holistic approach is essential, addressing not only substance use but also underlying risk factors such as adverse childhood experiences, mental health disorders particularly comorbid depression, anxiety, and other substance use disorders, and social inequities (138).

In addition, public health strategies must reflect cultural and regional variations in cannabis use patterns and product types. Tailored interventions should consider socioeconomic factors, particularly in Arab countries with legalized or regulated cannabis farming. In such contexts, policies must ensure that cannabis cultivation for medical or economic purposes does not inadvertently promote recreational use among youth. Interventions should also remain adaptable to emerging trends and substances of concern. This adaptability is crucial, as patterns of cannabis use are evolving, with increasing use of high-potency products, vaping, and both synthetic and semi-synthetic cannabinoids among youth (7, 140). Failure to update prevention strategies risks missing emerging forms of use or newly prevalent substances.

Given that spiritual interventions have been shown to be effective in supporting individuals with substance use problems (141), incorporating religious values into prevention efforts may enhance both community acceptance and intervention uptake, particularly in the Arab World, where religion plays a pivotal role in shaping norms and behaviors. Engaging faith leaders and religious institutions could reinforce anti-drug messaging, facilitate culturally congruent education, and help reduce the stigma associated with seeking help for substance use (142, 143).

Finally, future research should prioritize longitudinal studies to assess changes in cannabis use patterns over time and to clarify causal relationships between risk factors and consumption among youth. There is also a critical need to culturally validate assessment instruments in the Arab region, as many tools currently in use lack psychometric validation in this context (144). Strengthening measurement tools will improve the accuracy and comparability of findings and support the development of more effective, culturally appropriate interventions.

This systematic review has several limitations that should be acknowledged. First, many potentially relevant articles were inaccessible and, therefore, excluded. To minimize the risk of missing important data, the authors conducted a comprehensive literature search across multiple databases and grey literature sources, reviewed the references and citations of all included articles, and imposed no language restrictions. Second, the variation in how cannabis use was defined and the instruments used to measure it contributed to inconsistencies in the reported prevalence estimates, making it difficult to draw reliable comparisons between countries and settings. This lack of standardization in both conceptual and measurement approaches likely resulted in a wide range of findings. To enhance comparability, future studies should adopt standardized definitions and validated tools that accurately capture the extent of cannabis use among students in the Arab region. Third, as this review was limited to cross-sectional studies, it does not provide insights into the longitudinal progression or causality of cannabis use behaviors. Fourth, although the review provides valuable insights into cannabis use among students in schools and universities, it does not include other subgroups, such as out-of-school youth, dropouts, or refugees. These populations were intentionally excluded to maintain a consistent focus on individuals in formal educational settings. Including these groups could have introduced additional variability related to risk factors and living conditions, potentially affecting prevalence estimates. Nevertheless, these groups may be at a higher risk for cannabis use, underscoring the importance of future research specifically targeting these vulnerable populations. Lastly, the focus on the Arab World limits the generalizability of the findings to other regions with different sociocultural nuances and legal frameworks surrounding cannabis use. Future reviews might consider cross-regional comparisons to improve the understanding of cannabis consumption across diverse contexts.

In conclusion, this systematic review highlights the significant prevalence and correlates of cannabis use among school and university students across the Arab World. The findings indicate that cannabis consumption is a growing concern, especially among university students, influenced by a range of factors including country of residency, age, gender, family dynamics, and sociocultural beliefs. There is a dire need for targeted interventions that address modifiable risk factors, starting at the school level and extending into university settings, to efficiently mitigate the increasing trend of cannabis use among young people in the Arab region.

## Data Availability

The raw data supporting the conclusions of this article will be made available by the authors, without undue reservation.
