# Supplementary material for: Cannabis use among Arab students: a systematic review
Source: Front Psychiatry. 2025 Jul 10;16:1511563. doi: 10.3389/fpsyt.2025.1511563 (PMC12287767; doi:10.3389/fpsyt.2025.1511563)
Supplement: Supplementary file 1 [file Table1.docx]

Supplementary Material

# Supplementary Table 1: MeSH Terms.

**Cannabis**

| Mesh Terms | Entry Terms |
| --- | --- |
| Cannabis | CannabiHemp PlantHemp PlantsPlant, HempPlants, HempMarihuanaMarijuanaCannabis indicaCannabis sativaHempHempsHashishHashishsBhangBhangsGanjaGanjasCannabis |
| Marijuana abuse | Abuse, MarijuanaMarihuana AbuseAbuse, MarihuanaHashish AbuseAbuse, HashishCannabis-Related DisorderCannabis Related DisorderDisorder, Cannabis-RelatedCannabis AbuseAbuse, CannabisCannabis DependenceDependence, CannabisMarijuana DependenceDependence, MarijuanaMarijuana Abuse |
| Medical Marijuana | Marijuana, MedicalMarijuana TreatmentTreatment, MarijuanaMedical CannabisCannabis, MedicalMedicinal CannabisCannabis, MedicinalMedicinal MarijuanaMarijuana, MedicinalMarijuana DispensariesDispensaries, MarijuanaMedical Marijuana |
| Cannabinoid Hyperemesis Syndrome | Cannabinoid Hyperemesis SyndromesHyperemesis Syndrome, CannabinoidSyndrome, Cannabinoid HyperemesisCannabis Hyperemesis SyndromeCannabis Hyperemesis SyndromesHyperemesis Syndrome, CannabisSyndrome, Cannabis HyperemesisCannabinoid Hyperemesis Syndrome |
| Neodidymelliopsis cannabis | Phoma urticicolaPhoma cannabisDidymella urticicolaDidymella cannabisMycosphaerella cannabisNeodidymelliopsis cannabis |
| Marijuana Smoking | Marijuana SmokingSmoking, MarijuanaMarihuana SmokingSmoking, MarihuanaSmoking, BluntsBlunts SmokingBlunts SmokingsSmokings, BluntsSmoking BluntsBlunt, SmokingBlunts, SmokingSmoking BluntHashish SmokingSmoking, HashishCannabis SmokingSmoking, Cannabis |
| Xanthomonas cannabis | Xanthomonas cannabis |
| olivetolic acid cyclase, Cannabis sativa | olivetolic acid cyclase, Cannabis sativa |
| Can s 3 allergen, Cannabis sativa | Can s 3 allergen, Cannabis sativa |
| edestin protein, Cannabis sativa | edestin protein, Cannabis sativa |
| hempseed oil | hempseed oil |
| Dronabinol | Dronabinol |
| Marijuana Use | Marijuana useMarijuana UsesUse, MarijuanaUses, MarijuanaMarijuana-Related DisorderDisorder, Marijuana-RelatedDisorders, Marijuana-RelatedMarijuana Related DisorderMarijuana-Related DisordersRecreational Marijuana UseMarijuana Use, RecreationalMarijuana Uses, RecreationalRecreational Marijuana UsesUse, Recreational MarijuanaUses, Recreational Marijuana |
| Cannabinoids | CannabinoidCannabinoids |
|  | WeedPotTetrahydrocannabinolTHC |

**Arab World**

| Mesh Terms | Entry Terms |
| --- | --- |
| Arab World | Arab World |
| Arabs | ArabPalestiniansPalestinianBedouinsBedouinBedouin PeopleBedouin PeoplesPeople, BedouinArabs |
| United Arab Emirates | Trucial StatesAbu DhabiUnited Arab Emirates |
| Egypt | Arab Republic of EgyptUnited Arab RepublicEgypt |
| Rashaida people | Rashaida Arab peopleBani Rashid peopleRashaayda peopleRashaida people |
| Marsh Arab people | Marsh Arab people |
| Arab-Berber people | Arab-Berber people |
| Arab-Fulani people | Arab-Fulani people |
| Middle East | West BankNear EastGaza StripGaza Strip (Palestine)Middle East |
| Middle Eastern People | Middle Eastern PeoplesPeople, Middle EasternNear EasternersNear EasternerMiddle Eastern PersonMiddle Eastern PersonsPerson, Middle EasternSouthwestern Asian PersonAsian Person, SouthwesternPerson, Southwestern AsianSouthwestern Asian PersonsSouthwestern Asian PeopleAsian People, SouthwesternAsian Peoples, SouthwesternPeople, Southwestern AsianPeoples, Southwestern AsianSouthwestern Asian PeoplesMiddle EasternersMiddle EasternerSouthwestern AsiansAsian, SouthwesternMiddle Eastern People |
| Middle Eastern and North Africans | MENAsMiddle Eastern and North Africans |
| Lebanon | Lebanese RepublicLebanon |
| Syria | Syria |
| Iraq | Republic of IraqIraq |
| Algeria | Algeria |
| Bahrain | Bahrain |
| Jordan | Jordan |
| Jordanian people | Jordanian people |
| Kuwait | Kuwait |
| Libya | Libya |
| Mauritania | Mauritania |
| Morocco | MoroccoIfni |
| Oman | OmanMuscatMuscat and Oman |
| Qatar | KatarState of QatarQuatarQatar |
| Saudi Arabia | Kingdom of Saudi ArabiaSaudi Arabia |
| Sudan | Republic of the SudanSudan |
| South Sudan | South Sudan |
| Tunisia | Tunisia |
| Yemen | Republic of YemenDemocratic YemenSanaaNorth YemenAdenSouth YemenYemen |
| Comoros | Iles ComoresComoro IslandsMayotteComoros |
| Djibouti | Somaliland, FrenchRepublic of DjiboutiFrench SomalilandDjibouti |
| Somalia | Somalia |

**Students**

| **Mesh Terms** | **Entry Terms** |
| --- | --- |
| Students | Student  School Enrollment  Enrollment, School  Enrollments, School  School Enrollments  Students |
| Universities | Universities |
| Schools | School  Primary Schools  Primary School  School, Primary  Schools, Primary  Schools, Secondary  School, Secondary  Secondary School  Secondary Schools  Schools |
| - | College  Academy  Institute  University |
| - | Learner  Undergraduate  Freshman  Apprentice  Schoolchild  Undergrad  Observer  Registrant  Reader  Sophomore |

# Supplementary Table 2: Search Strategy.

| **PubMed** | ( ( “Cannabis” [Mesh Terms] OR “Marijuana abuse” [Mesh Terms] OR “Medical Marijuana” [Mesh Terms] OR “Cannabinoid Hyperemesis Syndrome” [Mesh Terms] OR “Neodidymelliopsis cannabis” [tiab:~0] OR “Marijuana Smoking” [Mesh Terms] OR “Xanthomonas cannabis” [tiab:~0] OR “olivetolic acid cyclase, Cannabis sativa” [tiab:~0] OR “Can s 3 allergen, Cannabis sativa” [tiab:~0] OR “edestin protein, Cannabis sativa” [tiab:~0] OR “hempseed oil” [tiab:~0] OR “Dronabinol” [Mesh Terms] OR “Marijuana Use” [Mesh Terms] OR “Cannabinoids” [Mesh Terms] OR “Cannabi” [All Fields] OR “Hemp Plant” [All Fields] OR “Hemp Plants” [All Fields] OR “Plant, Hemp” [All Fields] OR “Plants, Hemp” [All Fields] OR “Marihuana” [All Fields] OR “Marijuana” [All Fields] OR “Cannabis indica” [All Fields] OR “Cannabis sativa” [All Fields] OR “Hemp” [All Fields] OR “Hemps” [All Fields] OR “Hashish” [All Fields] OR “Bhang” [All Fields] OR “Ganja” [All Fields] OR “Cannabis” [All Fields] OR “Abuse, Marijuana” [All Fields] OR “Marihuana Abuse” [All Fields] OR “Abuse, Marihuana” [tiab:~0] OR “Hashish Abuse” [All Fields] OR “Abuse, Hashish” [All Fields] OR “Cannabis-Related Disorder” [All Fields] OR “Cannabis Related Disorder” [All Fields] OR “Disorder, Cannabis-Related” [All Fields] OR “Cannabis Abuse” [All Fields] OR “Abuse, Cannabis” [All Fields] OR “Cannabis Dependence” [All Fields] OR “Dependence, Cannabis” [All Fields] OR “Marijuana Dependence” [All Fields] OR “Dependence, Marijuana” [All Fields] OR “Marijuana Abuse” [All Fields] OR “Marijuana, Medical” [All Fields] OR “Marijuana Treatment” [All Fields] OR “Treatment, Marijuana” [All Fields] OR “Medical Cannabis” [All Fields] OR “Cannabis, Medical” [All Fields] OR “Medicinal Cannabis” [All Fields] OR “Cannabis, Medicinal” [All Fields] OR “Medicinal Marijuana” [All Fields] OR “Marijuana, Medicinal” [All Fields] OR “Marijuana Dispensaries” [All Fields] OR “Dispensaries, Marijuana” [tiab:~0] OR “Medical Marijuana” [All Fields] OR “Cannabinoid Hyperemesis Syndromes” [tiab:~0] OR “Hyperemesis Syndrome, Cannabinoid” [All Fields] OR “Syndrome, Cannabinoid Hyperemesis” [All Fields] OR “Cannabis Hyperemesis Syndrome” [All Fields] OR “Cannabis Hyperemesis Syndromes” [tiab:~0] OR “Hyperemesis Syndrome, Cannabis” [All Fields] OR “Syndrome, Cannabis Hyperemesis” [tiab:~0] OR “Cannabinoid Hyperemesis Syndrome” [All Fields] OR “Phoma urticicola” [tiab:~0] OR “Phoma cannabis” [tiab:~0] OR “Didymella urticicola” [tiab:~0] OR “Didymella cannabis” [tiab:~0] OR “Mycosphaerella cannabis” [tiab:~0] OR “Neodidymelliopsis cannabis” [tiab:~0] OR “Marijuana Smoking” [All Fields] OR “Smoking, Marijuana” [All Fields] OR “Marihuana Smoking” [All Fields] OR “Smoking, Marihuana” [All Fields] OR “Smoking, Blunts” [tiab:~0] OR “Blunts Smoking” [tiab:~0] OR “Blunts Smokings” [tiab:~0] OR “Smokings, Blunts” [tiab:~0] OR “Smoking Blunts” [All Fields] OR “Blunt, Smoking” [All Fields] OR “Blunts, Smoking” [All Fields] OR “Smoking Blunt” [tiab:~0] OR “Hashish Smoking” [All Fields] OR “Smoking, Hashish” [All Fields] OR “Cannabis Smoking” [All Fields] OR “Smoking, Cannabis” [All Fields] OR “Xanthomonas cannabis” [tiab:~0]OR “olivetolic acid cyclase, Cannabis sativa” [tiab:~0] OR “Can s 3 allergen, Cannabis sativa” [tiab:~0] OR “edestin protein, Cannabis sativa” [tiab:~0] OR “hempseed oil” [tiab:~0] OR “Dronabinol” [All Fields] OR “Marijuana use” [All Fields] OR “Marijuana Uses” [All Fields] OR “Use, Marijuana” [All Fields] OR “Uses, Marijuana” [All Fields] OR “Marijuana-Related Disorder” [tiab:~0] OR “Disorder, Marijuana-Related” [tiab:~0] OR “Disorders, Marijuana-Related” [All Fields] OR “Marijuana Related Disorder” [tiab:~0] OR “Marijuana-Related Disorders” [All Fields] OR “Recreational Marijuana Use” [All Fields] OR “Marijuana Use, Recreational” [tiab:~0] OR “Marijuana Uses, Recreational” [tiab:~0] OR “Recreational Marijuana Uses” [tiab:~0] OR “Use, Recreational Marijuana” [All Fields] OR “Uses, Recreational Marijuana” [tiab:~0] OR “Cannabinoid” [All Fields] OR “Cannabinoids” [All Fields] OR “Weed” [All Fields] OR “Pot” [All Fields] OR “Tetrahydrocannabinol” [All Fields] OR “THC” [All Fields] )  AND  ( ( “Arab World” [Mesh Terms] OR “Arabs” [Mesh Terms] OR “United Arab Emirates” [Mesh Terms] OR “Egypt” [Mesh Terms] OR “Rashaida people” [tiab:~0] OR “Marsh Arab people” [tiab:~0] OR “Arab-Berber people” [tiab:~0] OR “Arab-Fulani people” [tiab:~0] OR “Middle East” [Mesh Terms] OR “Middle Eastern People” [Mesh Terms] OR “Middle Eastern and North Africans” [Mesh Terms] OR “Lebanon” [Mesh Terms] OR “Syria” [Mesh Terms] OR “Iraq” [Mesh Terms] OR “Algeria” [Mesh Terms] OR “Bahrain” [Mesh Terms] OR “Jordan” [Mesh Terms] OR “Jordanian people” [All Fields] OR “Kuwait” [Mesh Terms] OR “Libya” [Mesh Terms] OR “Mauritania” [Mesh Terms] OR “Morocco” [Mesh Terms] OR “Oman” [Mesh Terms] OR “Qatar” [Mesh Terms] OR “Saudi Arabia” [Mesh Terms] OR “Sudan” [Mesh Terms] OR “South Sudan” [Mesh Terms] OR “Tunisia” [Mesh Terms] OR “Yemen” [Mesh Terms] OR “Comoros” [Mesh Terms] OR “Djibouti” [Mesh Terms] OR “Somalia” [Mesh Terms] OR “Arab World” [All Fields] OR “Arab” [All Fields] OR “Palestinians” [All Fields] OR “Palestinian” [All Fields] OR “Bedouins” [All Fields] OR “Bedouin” [All Fields] OR “Bedouin People” [All Fields] OR “Bedouin Peoples” [All Fields] OR “People, Bedouin” [tiab:~0] OR “Arabs” [All Fields] OR “Trucial States” [All Fields] OR “Abu Dhabi” [All Fields] OR “United Arab Emirates” [All Fields] OR “Arab Republic of Egypt” [All Fields] OR “United Arab Republic” [All Fields] OR “Egypt” [All Fields] OR “Rashaida Arab people” [tiab:~0] OR “Bani Rashid people” [tiab:~0] OR “Rashaayda people” [tiab:~0] OR “Rashaida people” [tiab:~0] OR “Marsh Arab people” [tiab:~0] OR “Arab-Berber people” [tiab:~0] OR “Arab-Fulani people” [tiab:~0] OR “West Bank” [All Fields] OR “Near East” [All Fields] OR “Gaza Strip” [All Fields] OR “Palestine ” [All Fields] OR “Middle East” [All Fields] OR “Middle Eastern Peoples” [All Fields] OR “People, Middle Eastern” [All Fields] OR “Near Easterners” [All Fields] OR “Near Easterner” [All Fields] OR “Middle Eastern Person” [tiab:~0] OR “Middle Eastern Persons” [All Fields] OR “Person, Middle Eastern” [tiab:~0] OR “Southwestern Asian Person” [tiab:~0] OR “Asian Person, Southwestern” [tiab:~0] OR “Person, Southwestern Asian” [tiab:~0] OR “Southwestern Asian Persons” [tiab:~0] OR “Southwestern Asian People” [tiab:~0] OR “Asian People, Southwestern” [tiab:~0] OR “Asian Peoples, Southwestern” [tiab:~0] OR “People, Southwestern Asian” [tiab:~0] OR “Peoples, Southwestern Asian” [tiab:~0] OR “Southwestern Asian Peoples” [tiab:~0] OR “Middle Easterners” [All Fields] OR “Middle Easterner” [All Fields] OR “Southwestern Asians” [tiab:~0] OR “Asian, Southwestern” [tiab:~0] OR “Middle Eastern People” [All Fields] OR “MENAs” [All Fields] OR “Middle Eastern and North Africans” [All Fields] OR “Lebanese Republic” [All Fields] OR “Lebanon” [All Fields] OR “Syria” [All Fields] OR “Republic of Iraq” [All Fields] OR “Iraq” [All Fields] OR “Algeria” [All Fields] OR “Bahrain” [All Fields] OR “Jordan” [All Fields] OR “Jordanian people” [tiab:~0] OR “Kuwait” [All Fields] OR “Libya” [All Fields] OR “Mauritania” [All Fields] OR “Morocco” [All Fields] OR “Ifni” [All Fields] OR “Oman” [All Fields] OR “Muscat” [All Fields] OR “Muscat and Oman” [tiab:~0] OR “Katar” [All Fields] OR “State of Qatar” [All Fields] OR “Quatar” [All Fields] OR “Qatar” [All Fields] OR “Kingdom of Saudi Arabia” [All Fields] OR “Saudi Arabia” [All Fields] OR “Republic of the Sudan” [All Fields] OR “Sudan” [All Fields] OR “South Sudan” [All Fields] OR “Tunisia” [All Fields] OR “Republic of Yemen” [All Fields] OR “Democratic Yemen” [All Fields] OR “Sanaa” [All Fields] OR “North Yemen” [All Fields] OR “Aden” [All Fields] OR “South Yemen” [All Fields] OR “Yemen” [All Fields] OR “Iles Comores” [tiab:~0] OR “Comoro Islands” [All Fields] OR “Mayotte” [All Fields] OR “Comoros” [All Fields] OR “Somaliland, French” [tiab:~0] OR “Republic of Djibouti” [All Fields] OR “French Somaliland” [All Fields] OR “Djibouti” [All Fields] OR “Somalia” [All Fields] )  AND  ( “Students” [Mesh Terms] OR “Universities” [Mesh Terms] OR “Schools” [Mesh Terms] OR “Student” [All Fields] OR “School Enrollment” [All Fields] OR “Enrollment, School” [All Fields] OR “Enrollments, School” [tiab:~0] OR “School Enrollments” [All Fields] OR “Students” [All Fields] OR “Universities” [All Fields] OR “School” [All Fields] OR “Primary Schools” [All Fields] OR “Primary School” [All Fields] OR “School, Primary” [All Fields] OR “Schools, Primary” [All Fields] OR “Schools, Secondary” [All Fields] OR “School, Secondary” [All Fields] OR “Secondary School” [All Fields] OR “Secondary Schools” [All Fields] OR “Schools” [All Fields] OR “College” [All Fields] OR “Academy” [All Fields] OR “Institute” [All Fields] OR “University” [All Fields] OR “Learner” [All Fields] OR “Undergraduate” [All Fields] OR “Freshman” [All Fields] OR “Apprentice” [All Fields] OR “Schoolchild” [All Fields] OR “Undergrad” [All Fields] OR “Observer” [All Fields] OR “Registrant” [All Fields] OR “Reader” [All Fields] OR “Sophomore” [All Fields] ) ) )  **Filter applied: Humans** | |
| --- | --- | --- |
| **Embase** | ( ( “Cannabis” OR “Marijuana abuse” OR “Medical Marijuana” OR “Cannabinoid Hyperemesis Syndrome” OR “Neodidymelliopsis cannabis” OR “Marijuana Smoking” OR “Xanthomonas cannabis” OR “olivetolic acid cyclase, Cannabis sativa” OR “Can s 3 allergen, Cannabis sativa” OR “edestin protein, Cannabis sativa” OR “hempseed oil” OR “Dronabinol” OR “Marijuana Use” OR “Cannabinoids” OR “Cannabi” OR “Hemp Plant” OR “Hemp Plants” OR “Plant, Hemp” OR “Plants, Hemp” OR “Marihuana” OR “Marijuana” OR “Cannabis indica” OR “Cannabis sativa” OR “Hemp” OR “Hemps” OR “Hashish” OR “Bhang” OR “Ganja” OR “Cannabis” OR “Abuse, Marijuana” OR “Marihuana Abuse” OR “Abuse, Marihuana” OR “Hashish Abuse” OR “Abuse, Hashish” OR “Cannabis-Related Disorder” OR “Cannabis Related Disorder” OR “Disorder, Cannabis-Related” OR “Cannabis Abuse” OR “Abuse, Cannabis” OR “Cannabis Dependence” OR “Dependence, Cannabis” OR “Marijuana Dependence” OR “Dependence, Marijuana” OR “Marijuana Abuse” OR “Marijuana, Medical” OR “Marijuana Treatment” OR “Treatment, Marijuana” OR “Medical Cannabis” OR “Cannabis, Medical” OR “Medicinal Cannabis” OR “Cannabis, Medicinal” OR “Medicinal Marijuana” OR “Marijuana, Medicinal” OR “Marijuana Dispensaries” OR “Dispensaries, Marijuana” OR “Medical Marijuana” OR “Cannabinoid Hyperemesis Syndromes” OR “Hyperemesis Syndrome, Cannabinoid” OR “Syndrome, Cannabinoid Hyperemesis” OR “Cannabis Hyperemesis Syndrome” OR “Cannabis Hyperemesis Syndromes” OR “Hyperemesis Syndrome, Cannabis” OR “Syndrome, Cannabis Hyperemesis” OR “Cannabinoid Hyperemesis Syndrome” OR “Phoma urticicola” OR “Phoma cannabis” OR “Didymella urticicola” OR “Didymella cannabis” OR “Mycosphaerella cannabis” OR “Neodidymelliopsis cannabis” OR “Marijuana Smoking” OR “Smoking, Marijuana” OR “Marihuana Smoking” OR “Smoking, Marihuana” OR “Smoking, Blunts” OR “Blunts Smoking” OR “Blunts Smokings” OR “Smokings, Blunts” OR “Smoking Blunts” OR “Blunt, Smoking” OR “Blunts, Smoking” OR “Smoking Blunt” OR “Hashish Smoking” OR “Smoking, Hashish” OR “Cannabis Smoking” OR “Smoking, Cannabis” OR “Xanthomonas cannabis” OR “olivetolic acid cyclase, Cannabis sativa” OR “Can s 3 allergen, Cannabis sativa” OR “edestin protein, Cannabis sativa” OR “hempseed oil” OR “Dronabinol” OR “Marijuana use” OR “Marijuana Uses” OR “Use, Marijuana” OR “Uses, Marijuana” OR “Marijuana-Related Disorder” OR “Disorder, Marijuana-Related” OR “Disorders, Marijuana-Related” OR “Marijuana Related Disorder” OR “Marijuana-Related Disorders” OR “Recreational Marijuana Use” OR “Marijuana Use, Recreational” OR “Marijuana Uses, Recreational” OR “Recreational Marijuana Uses” OR “Use, Recreational Marijuana” OR “Uses, Recreational Marijuana” OR “Cannabinoid” OR “Cannabinoids” OR “Weed” OR “Pot” OR “Tetrahydrocannabinol” OR “THC” )  AND  ( ( “Arab World” OR “Arabs” OR “United Arab Emirates” OR “Egypt” OR “Rashaida people” OR “Marsh Arab people” OR “Arab-Berber people” OR “Arab-Fulani people” OR “Middle East” OR “Middle Eastern People” OR “Middle Eastern and North Africans” OR “Lebanon” OR “Syria” OR “Iraq” OR “Algeria” OR “Bahrain” OR “Jordan” OR “Jordanian people” OR “Kuwait” OR “Libya” OR “Mauritania” OR “Morocco” OR “Oman” OR “Qatar” OR “Saudi Arabia” OR “Sudan” OR “South Sudan” OR “Tunisia” OR “Yemen” OR “Comoros” OR “Djibouti” OR “Somalia” OR “Arab World” OR “Arab” OR “Palestinians” OR “Palestinian” OR “Bedouins” OR “Bedouin” OR “Bedouin People” OR “Bedouin Peoples” OR “People, Bedouin” OR “Arabs” OR “Trucial States” OR “Abu Dhabi” OR “United Arab Emirates” OR “Arab Republic of Egypt” OR “United Arab Republic” OR “Egypt” OR “Rashaida Arab people” OR “Bani Rashid people” OR “Rashaayda people” OR “Rashaida people” OR “Marsh Arab people” OR “Arab-Berber people” OR “Arab-Fulani people” OR “West Bank” OR “Near East” OR “Gaza Strip” OR “Palestine ” OR “Middle East” OR “Middle Eastern Peoples” OR “People, Middle Eastern” OR “Near Easterners” OR “Near Easterner” OR “Middle Eastern Person” OR “Middle Eastern Persons” OR “Person, Middle Eastern” OR “Southwestern Asian Person” OR “Asian Person, Southwestern” OR “Person, Southwestern Asian” OR “Southwestern Asian Persons” OR “Southwestern Asian People” OR “Asian People, Southwestern” OR “Asian Peoples, Southwestern” OR “People, Southwestern Asian” OR “Peoples, Southwestern Asian” OR “Southwestern Asian Peoples” OR “Middle Easterners” OR “Middle Easterner” OR “Southwestern Asians” OR “Asian, Southwestern” OR “Middle Eastern People” OR “MENAs” OR “Middle Eastern and North Africans” OR “Lebanese Republic” OR “Lebanon” OR “Syria” OR “Republic of Iraq” OR “Iraq” OR “Algeria” OR “Bahrain” OR “Jordan” OR “Jordanian people” OR “Kuwait” OR “Libya” OR “Mauritania” OR “Morocco” OR “Ifni” OR “Oman” OR “Muscat” OR “Muscat and Oman” OR “Katar” OR “State of Qatar” OR “Quatar” OR “Qatar” OR “Kingdom of Saudi Arabia” OR “Saudi Arabia” OR “Republic of the Sudan” OR “Sudan” OR “South Sudan” OR “Tunisia” OR “Republic of Yemen” OR “Democratic Yemen” OR “Sanaa” OR “North Yemen” OR “Aden” OR “South Yemen” OR “Yemen” OR “Iles Comores” OR “Comoro Islands” OR “Mayotte” OR “Comoros” OR “Somaliland, French” OR “Republic of Djibouti” OR “French Somaliland” OR “Djibouti” OR “Somalia” )  AND  ( “Students” OR “Universities” OR “Schools” OR “Student” OR “School Enrollment” OR “Enrollment, School” OR “Enrollments, School” OR “School Enrollments” OR “Students” OR “Universities” OR “School” OR “Primary Schools” OR “Primary School” OR “School, Primary” OR “Schools, Primary” OR “Schools, Secondary” OR “School, Secondary” OR “Secondary School” OR “Secondary Schools” OR “Schools” OR “College” OR “Academy” OR “Institute” OR “University” OR “Learner” OR “Undergraduate” OR “Freshman” OR “Apprentice” OR “Schoolchild” OR “Undergrad” OR “Observer” OR “Registrant” OR “Reader” OR “Sophomore” ) ) )  **Filter applied: Humans** | |
| **Scopus** | ALL ( ( “Cannabis” OR “Marijuana abuse” OR “Medical Marijuana” OR “Cannabinoid Hyperemesis Syndrome” OR “Neodidymelliopsis cannabis” OR “Marijuana Smoking” OR “Xanthomonas cannabis” OR “olivetolic acid cyclase, Cannabis sativa” OR “Can s 3 allergen, Cannabis sativa” OR “edestin protein, Cannabis sativa” OR “hempseed oil” OR “Dronabinol” OR “Marijuana Use” OR “Cannabinoids” OR “Cannabi” OR “Hemp Plant” OR “Hemp Plants” OR “Plant, Hemp” OR “Plants, Hemp” OR “Marihuana” OR “Marijuana” OR “Cannabis indica” OR “Cannabis sativa” OR “Hemp” OR “Hemps” OR “Hashish” OR “Bhang” OR “Ganja” OR “Cannabis” OR “Abuse, Marijuana” OR “Marihuana Abuse” OR “Abuse, Marihuana” OR “Hashish Abuse” OR “Abuse, Hashish” OR “Cannabis-Related Disorder” OR “Cannabis Related Disorder” OR “Disorder, Cannabis-Related” OR “Cannabis Abuse” OR “Abuse, Cannabis” OR “Cannabis Dependence” OR “Dependence, Cannabis” OR “Marijuana Dependence” OR “Dependence, Marijuana” OR “Marijuana Abuse” OR “Marijuana, Medical” OR “Marijuana Treatment” OR “Treatment, Marijuana” OR “Medical Cannabis” OR “Cannabis, Medical” OR “Medicinal Cannabis” OR “Cannabis, Medicinal” OR “Medicinal Marijuana” OR “Marijuana, Medicinal” OR “Marijuana Dispensaries” OR “Dispensaries, Marijuana” OR “Medical Marijuana” OR “Cannabinoid Hyperemesis Syndromes” OR “Hyperemesis Syndrome, Cannabinoid” OR “Syndrome, Cannabinoid Hyperemesis” OR “Cannabis Hyperemesis Syndrome” OR “Cannabis Hyperemesis Syndromes” OR “Hyperemesis Syndrome, Cannabis” OR “Syndrome, Cannabis Hyperemesis” OR “Cannabinoid Hyperemesis Syndrome” OR “Phoma urticicola” OR “Phoma cannabis” OR “Didymella urticicola” OR “Didymella cannabis” OR “Mycosphaerella cannabis” OR “Neodidymelliopsis cannabis” OR “Marijuana Smoking” OR “Smoking, Marijuana” OR “Marihuana Smoking” OR “Smoking, Marihuana” OR “Smoking, Blunts” OR “Blunts Smoking” OR “Blunts Smokings” OR “Smokings, Blunts” OR “Smoking Blunts” OR “Blunt, Smoking” OR “Blunts, Smoking” OR “Smoking Blunt” OR “Hashish Smoking” OR “Smoking, Hashish” OR “Cannabis Smoking” OR “Smoking, Cannabis” OR “Xanthomonas cannabis” OR “olivetolic acid cyclase, Cannabis sativa” OR “Can s 3 allergen, Cannabis sativa” OR “edestin protein, Cannabis sativa” OR “hempseed oil” OR “Dronabinol” OR “Marijuana use” OR “Marijuana Uses” OR “Use, Marijuana” OR “Uses, Marijuana” OR “Marijuana-Related Disorder” OR “Disorder, Marijuana-Related” OR “Disorders, Marijuana-Related” OR “Marijuana Related Disorder” OR “Marijuana-Related Disorders” OR “Recreational Marijuana Use” OR “Marijuana Use, Recreational” OR “Marijuana Uses, Recreational” OR “Recreational Marijuana Uses” OR “Use, Recreational Marijuana” OR “Uses, Recreational Marijuana” OR “Cannabinoid” OR “Cannabinoids” OR “Weed” OR “Pot” OR “Tetrahydrocannabinol” OR “THC” )  AND  ( ( “Arab World” OR “Arabs” OR “United Arab Emirates” OR “Egypt” OR “Rashaida people” OR “Marsh Arab people” OR “Arab-Berber people” OR “Arab-Fulani people” OR “Middle East” OR “Middle Eastern People” OR “Middle Eastern and North Africans” OR “Lebanon” OR “Syria” OR “Iraq” OR “Algeria” OR “Bahrain” OR “Jordan” OR “Jordanian people” OR “Kuwait” OR “Libya” OR “Mauritania” OR “Morocco” OR “Oman” OR “Qatar” OR “Saudi Arabia” OR “Sudan” OR “South Sudan” OR “Tunisia” OR “Yemen” OR “Comoros” OR “Djibouti” OR “Somalia” OR “Arab World” OR “Arab” OR “Palestinians” OR “Palestinian” OR “Bedouins” OR “Bedouin” OR “Bedouin People” OR “Bedouin Peoples” OR “People, Bedouin” OR “Arabs” OR “Trucial States” OR “Abu Dhabi” OR “United Arab Emirates” OR “Arab Republic of Egypt” OR “United Arab Republic” OR “Egypt” OR “Rashaida Arab people” OR “Bani Rashid people” OR “Rashaayda people” OR “Rashaida people” OR “Marsh Arab people” OR “Arab-Berber people” OR “Arab-Fulani people” OR “West Bank” OR “Near East” OR “Gaza Strip” OR “Palestine ” OR “Middle East” OR “Middle Eastern Peoples” OR “People, Middle Eastern” OR “Near Easterners” OR “Near Easterner” OR “Middle Eastern Person” OR “Middle Eastern Persons” OR “Person, Middle Eastern” OR “Southwestern Asian Person” OR “Asian Person, Southwestern” OR “Person, Southwestern Asian” OR “Southwestern Asian Persons” OR “Southwestern Asian People” OR “Asian People, Southwestern” OR “Asian Peoples, Southwestern” OR “People, Southwestern Asian” OR “Peoples, Southwestern Asian” OR “Southwestern Asian Peoples” OR “Middle Easterners” OR “Middle Easterner” OR “Southwestern Asians” OR “Asian, Southwestern” OR “Middle Eastern People” OR “MENAs” OR “Middle Eastern and North Africans” OR “Lebanese Republic” OR “Lebanon” OR “Syria” OR “Republic of Iraq” OR “Iraq” OR “Algeria” OR “Bahrain” OR “Jordan” OR “Jordanian people” OR “Kuwait” OR “Libya” OR “Mauritania” OR “Morocco” OR “Ifni” OR “Oman” OR “Muscat” OR “Muscat and Oman” OR “Katar” OR “State of Qatar” OR “Quatar” OR “Qatar” OR “Kingdom of Saudi Arabia” OR “Saudi Arabia” OR “Republic of the Sudan” OR “Sudan” OR “South Sudan” OR “Tunisia” OR “Republic of Yemen” OR “Democratic Yemen” OR “Sanaa” OR “North Yemen” OR “Aden” OR “South Yemen” OR “Yemen” OR “Iles Comores” OR “Comoro Islands” OR “Mayotte” OR “Comoros” OR “Somaliland, French” OR “Republic of Djibouti” OR “French Somaliland” OR “Djibouti” OR “Somalia” )  AND  ( “Students” OR “Universities” OR “Schools” OR “Student” OR “School Enrollment” OR “Enrollment, School” OR “Enrollments, School” OR “School Enrollments” OR “Students” OR “Universities” OR “School” OR “Primary Schools” OR “Primary School” OR “School, Primary” OR “Schools, Primary” OR “Schools, Secondary” OR “School, Secondary” OR “Secondary School” OR “Secondary Schools” OR “Schools” OR “College” OR “Academy” OR “Institute” OR “University” OR “Learner” OR “Undergraduate” OR “Freshman” OR “Apprentice” OR “Schoolchild” OR “Undergrad” OR “Observer” OR “Registrant” OR “Reader” OR “Sophomore” ) ) )  **Filter applied: Humans**  **Filter applied: TITLE-ABS-KEY** | |
| **PsycInfo** | **S3** | #S1 AND #S2  **Filter applied: Humans** |
|  | **S2** | All Text  ( Arab World OR Arabs OR United Arab Emirates OR Egypt OR Rashaida people OR Marsh Arab people OR Arab-Berber people OR Arab-Fulani people OR Middle East OR Middle Eastern People OR Middle Eastern and North Africans OR Lebanon OR Syria OR Iraq OR Algeria OR Bahrain OR Jordan OR Jordanian people OR Kuwait OR Libya OR Mauritania OR Morocco OR Oman OR Qatar OR Saudi Arabia OR Sudan OR South Sudan OR Tunisia OR Yemen OR Comoros OR Djibouti OR Somalia OR Arab World OR Arab OR Palestinians OR Palestinian OR Bedouins OR Bedouin OR Bedouin People OR Bedouin Peoples OR People, Bedouin OR Arabs OR Trucial States OR Abu Dhabi OR United Arab Emirates OR Arab Republic of Egypt OR United Arab Republic OR Egypt OR Rashaida Arab people OR Bani Rashid people OR Rashaayda people OR Rashaida people OR Marsh Arab people OR Arab-Berber people OR Arab-Fulani people OR West Bank OR Near East OR Gaza Strip OR Palestine OR Middle East OR Middle Eastern Peoples OR People, Middle Eastern OR Near Easterners OR Near Easterner OR Middle Eastern Person OR Middle Eastern Persons OR Person, Middle Eastern OR Southwestern Asian Person OR Asian Person, Southwestern OR Person, Southwestern Asian OR Southwestern Asian Persons OR Southwestern Asian People OR Asian People, Southwestern OR Asian Peoples, Southwestern OR People, Southwestern Asian OR Peoples, Southwestern Asian OR Southwestern Asian Peoples OR Middle Easterners OR Middle Easterner OR Southwestern Asians OR Asian, Southwestern OR Middle Eastern People OR MENAs OR Middle Eastern and North Africans OR Lebanese Republic OR Lebanon OR Syria OR Republic of Iraq OR Iraq OR Algeria OR Bahrain OR Jordan OR Jordanian people OR Kuwait OR Libya OR Mauritania OR Morocco OR Ifni OR Oman OR Muscat OR Muscat and Oman OR Katar OR State of Qatar OR Quatar OR Qatar OR Kingdom of Saudi Arabia OR Saudi Arabia OR Republic of the Sudan OR Sudan OR South Sudan OR Tunisia OR Republic of Yemen OR Democratic Yemen OR Sanaa OR North Yemen OR Aden OR South Yemen OR Yemen OR Iles Comores OR Comoro Islands OR Mayotte OR Comoros OR Somaliland, French OR Republic of Djibouti OR French Somaliland OR Djibouti OR Somalia )  AND ( Students OR Universities OR Schools OR Student OR School Enrollment OR Enrollment, School OR Enrollments, School OR School Enrollments OR Students OR Universities OR School OR Primary Schools OR Primary School OR School, Primary OR Schools, Primary OR Schools, Secondary OR School, Secondary OR Secondary School OR Secondary Schools OR Schools OR College OR Academy OR Institute OR University OR Learner OR Undergraduate OR Freshman OR Apprentice OR Schoolchild OR Undergrad OR Observer OR Registrant OR Reader OR Sophomore ) |
|  | **S1** | All Text:  ( Cannabis OR Marijuana abuse OR Medical Marijuana OR Cannabinoid Hyperemesis Syndrome OR Neodidymelliopsis cannabis OR Marijuana Smoking OR Xanthomonas cannabis OR olivetolic acid cyclase, Cannabis sativa OR Can s 3 allergen, Cannabis sativa OR edestin protein, Cannabis sativa OR hempseed oil OR Dronabinol OR Marijuana Use OR Cannabinoids OR Cannabi OR Hemp Plant OR Hemp Plants OR Plant, Hemp OR Plants, Hemp OR Marihuana OR Marijuana OR Cannabis indica OR Cannabis sativa OR Hemp OR Hemps OR Hashish OR Bhang OR Ganja OR Cannabis OR Abuse, Marijuana OR Marihuana Abuse OR Abuse, Marihuana OR Hashish Abuse OR Abuse, Hashish OR Cannabis-Related Disorder OR Cannabis Related Disorder OR Disorder, Cannabis-Related OR Cannabis Abuse OR Abuse, Cannabis OR Cannabis Dependence OR Dependence, Cannabis OR Marijuana Dependence OR Dependence, Marijuana OR Marijuana Abuse OR Marijuana, Medical OR Marijuana Treatment OR Treatment, Marijuana OR Medical Cannabis OR Cannabis, Medical OR Medicinal Cannabis OR Cannabis, Medicinal OR Medicinal Marijuana OR Marijuana, Medicinal OR Marijuana Dispensaries OR Dispensaries, Marijuana OR Medical Marijuana OR Cannabinoid Hyperemesis Syndromes OR Hyperemesis Syndrome, Cannabinoid OR Syndrome, Cannabinoid Hyperemesis OR Cannabis Hyperemesis Syndrome OR Cannabis Hyperemesis Syndromes OR Hyperemesis Syndrome, Cannabis OR Syndrome, Cannabis Hyperemesis OR Cannabinoid Hyperemesis Syndrome OR Phoma urticicola OR Phoma cannabis OR Didymella urticicola OR Didymella cannabis OR Mycosphaerella cannabis OR Neodidymelliopsis cannabis OR Marijuana Smoking OR Smoking, Marijuana OR Marihuana Smoking OR Smoking, Marihuana OR Smoking, Blunts OR Blunts Smoking OR Blunts Smokings OR Smokings, Blunts OR Smoking Blunts OR Blunt, Smoking OR Blunts, Smoking OR Smoking Blunt OR Hashish Smoking OR Smoking, Hashish OR Cannabis Smoking OR Smoking, Cannabis OR Xanthomonas cannabis OR olivetolic acid cyclase, Cannabis sativa OR Can s 3 allergen, Cannabis sativa OR edestin protein, Cannabis sativa OR hempseed oil OR Dronabinol OR Marijuana use OR Marijuana Uses OR Use, Marijuana OR Uses, Marijuana OR Marijuana-Related Disorder OR Disorder, Marijuana-Related OR Disorders, Marijuana-Related OR Marijuana Related Disorder OR Marijuana-Related Disorders OR Recreational Marijuana Use OR Marijuana Use, Recreational OR Marijuana Uses, Recreational OR Recreational Marijuana Uses OR Use, Recreational Marijuana OR Uses, Recreational Marijuana OR Cannabinoid OR Cannabinoids OR Weed OR Pot OR Tetrahydrocannabinol OR THC ) |

# Supplementary Table 3: Title and Abstract Screening Guide.

| **Record number** | **Published 2000 or later** | **Cross-sectional study** | **Arab student population** | **Cannabis mentioned as outcome** | **Include?*** |
| --- | --- | --- | --- | --- | --- |
|  | ☐ Yes ☐ No | ☐ Yes ☐ No | ☐ Yes ☐ No | ☐ Yes ☐ No | ☐ Yes ☐ No |

*Instructions: Reviewers should independently assess each criterion and record their inclusion decision in the final column.

# Supplementary Table 4: Full Text Screening Guide and Reasons for Exclusion.

| **Record number** | **Not school or university population** | **Not Arab participants (or not stratified as Arab vs. non-Arab)** | **Not cross-sectional design** | **Cannabis use not assessed/ measured** | **No access to full text** | **Include in review?*** | **Comments** |
| --- | --- | --- | --- | --- | --- | --- | --- |
|  | ☐ | ☐ | ☐ | ☐ | ☐ | ☐ Yes  ☐ No |  |

*Reviewers should independently assess each criterion and record their decision in the *Include in review?* column. If any exclusion box is ticked, the study is excluded. Reasons for exclusion should be recorded and tabulated.

# Supplementary Table 5: NIH Quality Assessment Tool for Observational Cohort and Cross-Sectional Studies.

# Individual item-level assessments for each included study (n = 48) were conducted using this tool. These data are available from the authors upon request.

| **Question** | **Yes** | **No** | **Other (CD, NR, NA)** | **QR (Good, Fair, Poor)** |
| --- | --- | --- | --- | --- |
| 1. Was the research question or objective in this paper clearly stated? |  |  |  |  |
| 2. Was the study population clearly specified and defined? |  |  |  |  |
| 3. Was the participation rate of eligible persons at least 50%? |  |  |  |  |
| 4. Were all the subjects selected or recruited from the same or similar populations (including the same time period)? Were inclusion and exclusion criteria for being in the study prespecified and applied uniformly to all participants? |  |  |  |  |
| 5. Was a sample size justification, power description, or variance and effect estimates provided? |  |  |  |  |
| 6. For the analyses in this paper, were the exposure(s) of interest measured prior to the outcome(s) being measured? |  |  |  |  |
| 7. Was the timeframe sufficient so that one could reasonably expect to see an association between exposure and outcome if it existed? |  |  |  |  |
| 8. For exposures that can vary in amount or level, did the study examine different levels of the exposure as related to the outcome (e.g., categories of exposure, or exposure measured as continuous variable)? |  |  |  |  |
| 9. Were the exposure measures (independent variables) clearly defined, valid, reliable, and implemented consistently across all study participants? |  |  |  |  |
| 10. Was the exposure(s) assessed more than once over time? |  |  |  |  |
| 11. Were the outcome measures (dependent variables) clearly defined, valid, reliable, and implemented consistently across all study participants? |  |  |  |  |
| 12. Were the outcome assessors blinded to the exposure status of participants? |  |  |  |  |
| 13. Was loss to follow-up after baseline 20% or less? |  |  |  |  |
| 14. Were key potential confounding variables measured and adjusted statistically for their impact on the relationship between exposure(s) and outcome(s)? |  |  |  |  |

*CD: cannot determine, NR: not reported, NA: not applicable, QR: quality rating.
